# Morphological and Physiological Traits Associated with Yield under Reduced Irrigation in Chilean Coastal Lowland Quinoa

**DOI:** 10.3390/plants11030323

**Published:** 2022-01-26

**Authors:** Kathryn Dumschott, Nathalie Wuyts, Christian Alfaro, Dalma Castillo, Fabio Fiorani, Andrés Zurita-Silva

**Affiliations:** 1 Institute for Biology I, BioSC, RWTH Aachen University, 52056 Aachen, Germany; k.dumschott@fz-juelich.de; 2 Institute of Bio- and Geosciences, Bioinformatics (IBG-4), Forschungszentrum Jülich GmbH, 52425 Jülich, Germany; 3 Institute of Bio- and Geosciences, Plant Sciences (IBG-2), Forschungszentrum Jülich GmbH, 52425 Jülich, Germany; n.wuyts@fz-juelich.de; 4 Centro de Investigación Intihuasi (AZS), Instituto de Investigaciones Agropecuarias, La Serena 1722093, Chile; calfaro@inia.cl (C.A.); dalma.castillo@inia.cl (D.C.); 5 Centro de Investigación Rayentué (CA), Instituto de Investigaciones Agropecuarias, Rengo 2940000, Chile; 6 Centro de Investigación Quilamapu, Instituto de Investigaciones Agropecuarias, Chillán 3780000, Chile

**Keywords:** *Chenopodium quinoa* Willd., field trial, hyperspectral imaging, phenotyping, quinoa, reduced irrigation, thermal imaging, yield

## Abstract

Quinoa (*Chenopodium quinoa* Willd.) is a genetically diverse crop that has gained popularity in recent years due to its high nutritional content and ability to tolerate abiotic stresses such as salinity and drought. Varieties from the coastal lowland ecotype are of particular interest due to their insensitivity to photoperiod and their potential to be cultivated in higher latitudes. We performed a field experiment in the southern Atacama Desert in Chile to investigate the responses to reduced irrigation of nine previously selected coastal lowland self-pollinated (CLS) lines and the commercial cultivar Regalona. We found that several lines exhibited a yield and seed size superior to Regalona, also under reduced irrigation. Plant productivity data were analyzed together with morphological and physiological traits measured at the visible inflorescence stage to estimate the contribution of these traits to differences between the CLS lines and Regalona under full and reduced irrigation. We applied proximal sensing methods and found that thermal imaging provided a promising means to estimate variation in plant water use relating to yield, whereas hyperspectral imaging separated lines in a different way, potentially related to photosynthesis as well as water use.

## 1. Introduction

Quinoa (*Chenopodium quinoa* Willd.) is a highly nutritious member of the Amaranthaceae, originating from the Andean region of Central and South America. Originally cultivated by the Incas during pre-Colombian times [1], quinoa became a staple food of the Incan Empire and is now considered an important food crop in many South American countries [2]. Quinoa grains are gluten free and highly nutritious, containing high-quality protein and all essential amino acids, vitamins, minerals and antioxidants (flavonoids and polyphenols) that contribute to the health-promoting effects of this food crop [3,4,5,6,7,8,9]. Additionally, the seeds exhibit a high content of unsaturated fatty acids (oleic, linoleic, and α-linolenic acids) and a close to optimal omega-6/omega-3 ratio, which support the oil quality of this crop [10]. On the other hand, the seeds accumulate saponins, commonly considered anti-nutritional factors due to their hemolytic, membranolytic, and fungitoxic activities [11]. In recent decades, quinoa has become a target of research worldwide due to its potential contribution to food security [2,12]. Quinoa is a genetically diverse crop that thrives in the heterogeneous environments of the Andean region from which it originated [13]. Quinoa genotypes, landraces and cultivars can be classified into five ecotypes, which exhibit wide ranges of adaptations to elevation, annual rainfall, soil fertility, temperature and photoperiod [2].

Quinoa is also known for its ability to grow in marginal environments and tolerate a range of adverse growth conditions, such as high salinity [14,15,16,17], heat [18] and drought [15,19,20]. Detailed studies of quinoa subjected to drought stress have been conducted, both in the field and in greenhouse experiments, providing insight into the key physiological adaptations of quinoa. Increased water use efficiency associated with abscisic acid (ABA)-induced stomatal closure [21,22,23,24] is a common strategy implemented by quinoa in response to drought stress. Leaf ABA concentration has been observed to accumulate in response to increased stress in the field, suggesting it is an important mechanism for drought tolerance in quinoa [21]. In addition to ABA signaling, hydraulic regulation through changes in turgor may play an equally important role in stomatal closure for certain quinoa varieties subject to water stress [25]. Another strategy is the induction of metabolic adaptations that improve tolerance to osmotic or water stress, and which involve an increased synthesis of osmoprotectants such as free amino acids, proline, and soluble sugars (glucose, trehalose), to enhance scavenging of reactive oxygen species and to protect plants from destructive oxidative reactions [26,27]. Reductions in photosynthetic rates and the efficiency of photosystem II have also been observed in quinoa plants in response to water deficit [24,28]. Strategies used to mitigate water deficit stress have been shown to differ between quinoa varieties depending on their geographical origin. In a study involving two quinoa varieties, Sun et al. [23] observed that the relatively slower growth rates and smaller leaf areas of varieties originating from adverse environments were more effective at tolerating drought stress compared to fast-growing varieties originating from nutrient-rich environments, due to reduced overall transpiration and water loss [23].

To date, most drought stress studies involving quinoa have focused on only a few bred varieties and seldom compare ecotypes or ecotype-derived self-pollinated progenies. The diversity of quinoa and the establishment of new breeding and improvement programs to develop new varieties better adapted to different environmental conditions remain largely unexplored [13,29,30,31]. To this end, unique germplasm collections representing local and regional biodiversity are of particular interest as a source of variation. One major consideration when selecting quinoa material for this study was the sensitivity of quinoa to photoperiod, as this trait can significantly limit quinoa adaptation and breeding efforts in high-latitude regions, such as Europe [29,32]. Disruption to seed filling and maturation, resulting in continued vegetative growth and flowering, has been observed in photoperiod-sensitive lines grown in photoperiods of longer than 12 h [29,32]. Quinoa cultivars from the coastal lowland ecotype show an insensitivity to photoperiod, as they are adapted to the coastal conditions of southern Chile (latitudes up to ~40° S) and have already been used for European-bred cultivars from Denmark and the Netherlands [29,33,34]. Therefore, we focused on the coastal lowland ecotype and selected lines from the INIA SeedBank collection (Chile), based on morphology and yield observed in the field and further developed in a breeding program run by INIA Chile.

The physiological characterization and classification of lines according to their tolerance to drought stress customarily involves destructive or laborious measurements of traits such as leaf water potential or photosynthesis and stomatal conductance. These methods are often time-consuming and difficult to apply on a large scale. Screening of genotypes for pre-breeding can be facilitated by using more high-throughput phenotyping methods to measure photosynthetic status, spectral reflectance and canopy temperature [35,36]. These methods, in particular vegetation indices based on spectral reflectance measurements, have recently begun to be applied in quinoa research on drought stress [37,38].

In this study, we implemented a combination of standard and more recent approaches to measure productivity and underlying morphological and physiological traits of nine novel Chilean coastal lowland (CLS) lines and one commercial cultivar, Regalona Baer (hereafter referred to as cv Regalona), grown in a field experiment subject to a full and reduced irrigation regime starting from the branching stage (extended BBCH 20 [39]). Standard approaches included the non-invasive measurement of chlorophyll fluorescence and destructive measurements of water potential, relative water content, and shoot morphology. These were tested alongside recent approaches which can be scaled to high throughput, including hyperspectral and thermal imaging. All measurements were taken at the visible inflorescence stage of development (extended BBCH 59 [39]) and compared to seed yield and seed size determined at physiological maturity, as well as plant development over the course of the trial. We aimed to determine differences in yield and seed size among the selected CLS lines compared to cv Regalona, and to detect the underlying morphological and physiological traits that may contribute to yield and seed size determination under full and reduced irrigation. A better understanding of how well-established and more recently developed phenotyping methods can be deployed to determine traits contributing to yield under adverse conditions will help improve the effectiveness of future quinoa breeding programs and crop management.

## 2. Results

### 2.1. Agronomical Traits at Harvest

**Figure 1:**
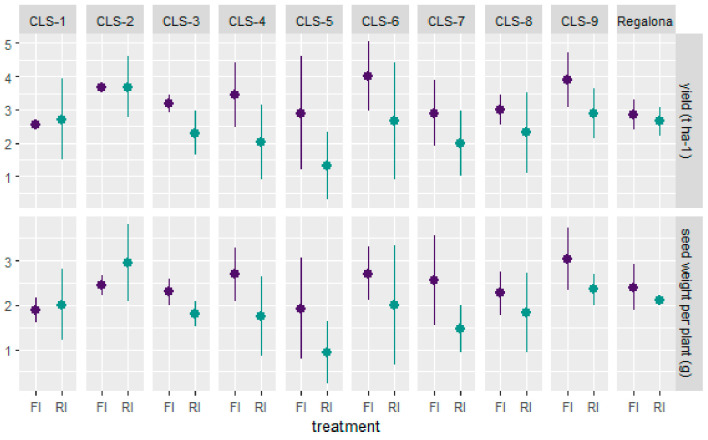
Productivity of nine coastal lowland self-pollinated lines (CLS) and commercial cv Regalona in a field experiment under full irrigation (FI, purple) and reduced irrigation (RI, green). Productivity is presented as yield expressed in t ha^−1^ (**top** panel) and the seed weight per plant (**bottom** panel) collected in 1 m of two central rows per plot. Data are presented as the mean (dot) and the standard deviation around the mean (vertical bar).

A significant treatment effect was observed for plant yield (*p* < 0.01), with plants receiving reduced irrigation (RI) yielding less than fully irrigated (FI) plants (Figure 1). There was no significant genotype or genotype by treatment interaction effect. However, lines CLS-1 and CLS-2 sustained a comparable yield despite a reduced water supply, which was similar to the response observed for the commercial cv Regalona. In the other CLS lines, the yield was decreased by water deficit (Figure 1). When considering seed weight per plant, treatment was again the only significant factor (*p* < 0.01), with average seed weights of 2.42 g and 1.92 g for FI and RI plants, respectively. Interestingly, lines CLS-1 and CLS-2 increased their average seed weight per plant in response to RI (1.88 g vs. 2.01 g for CLS-1 FI vs. RI samples, and 2.45 g vs. 2.94 g for CLS-2 FI vs. RI samples) (Figure 1).

For the 1000 seed weight, both the treatment (*p* < 0.0001) and genotype effects (*p* < 0.0001) were significant, but not the genotype by treatment interaction effect. The average 1000 seed weight was 3.08 g for FI plants and 2.86 g for RI plants (Table 1). The highest weights corresponded to lines CLS-3 and CLS-7, whereas the lowest weights were recorded in lines CLS-8, CLS-6 and cv Regalona. It is noteworthy that among the highest 1000 seed weights, CLS-7 was not affected by the RI treatment (Figure S1).

Significant differences at both the treatment (*p* < 0.05) and genotype (*p* < 0.0001) level were found for the 1.7 mm-caliber seed weight. The average weight for the FI treatment was 4.82 g, whereas the RI average was significantly lower (3.85 g). The highest 1.7 mm-caliber seed size weight was observed for line CLS-1, followed by CLS-3 and CLS-7 (Table 1). Interestingly, these three lines did not show similar decreases in the RI treatment, but for CLS-3 and CLS-7 the average difference between treatments was comparatively small (Figure S1). When also considering lower caliber seeds (1.4 mm and 1.18 mm), seed size displayed an inverse effect. A decrease in the proportion of higher caliber seeds occurred simultaneously with an increase in the proportion of lower caliber seeds (Figure S1).

For panicle length, we found a significant effect of genotype (*p* < 0.0001) and genotype by treatment interaction (*p* < 0.0001). Within the FI treatment, CLS-2 and cv Regalona had the shortest panicles, followed by CLS-7 and CLS-9, whereas lines CLS-1, CLS-5, CLS-6 and CLS-8 had the longest panicles. In the RI treatment, CLS-5 still had the largest panicle length, followed by CLS-1, CLS-6 and CLS-8. The other CLS lines and cv Regalona all had shorter panicles. Panicle width showed a significant treatment (*p* < 0.01), genotype (*p* < 0.0001) and genotype by treatment interaction effect (*p* < 0.0001). The panicle width was reduced in the RI treatment for lines CLS-2, CLS-3, CLS-4 and CLS-9 (Figure S2b). Within the FI treatment, panicles were smallest in width in CLS-7 and largest in CLS-6, CLS-8 and CLS-9. The widest panicles in the RI treatment were found for lines CLS-6, CLS-8 and CLS-5. The lines CLS-6 and CLS-8 had the overall largest panicles, together with CLS-5 whose panicles even increased in size under RI.

**Figure 2:**
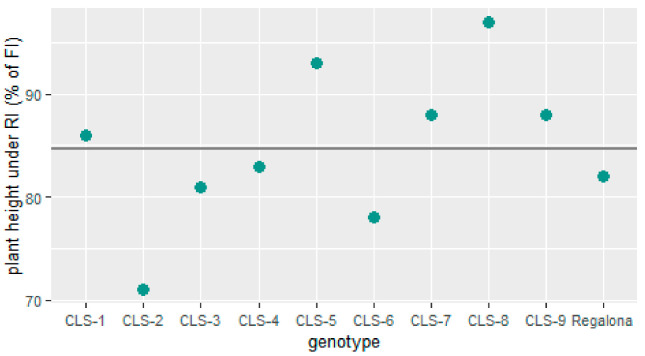
Plant height at harvest under reduced irrigation (RI) expressed as a percentage of plant height under full irrigation (FI) for nine coastal lowland self-pollinated lines (CLS) and commercial cv Regalona. The mean plant height under RI as a % of FI is indicated by a horizontal line.

The plant height at harvest ranged from 99 to 127 cm (with an average height of 115 cm) in the FI treatment, and from 87 to 108 cm (with an average height of 97 cm) in the RI treatment (Figure S3a). For all genotypes except CLS-5 and CLS-8, plant height was significantly (*p* < 0.01) reduced under RI conditions (Figure S3b). The largest effect of RI on plant height was observed for CLS-2 and CLS-6, whereas the effect was small for CLS-8 and CLS-5 (Figure 2). Plant height recorded for line CLS-8 was relatively short in both treatments, while CLS-5 plants were considered medium height when compared to the other lines (Figure S3). The tallest lines at harvest were CLS-1, CLS-3, CLS-4, CLS-6 and CLS-7, whereas plants of CLS-9 and cv Regalona were the shortest in both treatments.

Yield responses to reduced water availability in a genotype panel can also be assessed by means of drought and yield tolerance indices. These are based on the overall effect on yield or by comparing genotypes of interest to the yield reduction in a set of reference genotypes [40]. The drought tolerance index (DTI) was 0.76 in our field trial involving nine CLS lines and the commercial cv Regalona as a reference. Therefore, an overall 24% yield loss occurred under RI conditions. The DTI of CLS-1 and CLS-2 was the highest (1.41 and 1.32, respectively), as no yield loss was recorded under RI, while cv Regalona had a DTI of 1.23 and was also relatively unaffected by drought (Table 2). Among the other lines, CLS-5 was the most affected by drought with a DTI of 0.60. In this field experiment, all CLS lines, except CLS-1 and CLS-2, performed worse than cv Regalona. As recommended by Ober et al. and del Pozo et al. [40,41], we also calculated the yield tolerance index (YTI) because it may differentiate between genotypes that perform better under drought stress due to an inherently high yield potential, and those that intrinsically have greater drought tolerance. This difference was observed in our field trial for CLS-1 and CLS-2, which had YTI’s of 0.66 and 1.29, respectively (Table 2). CLS-2 was therefore a high yielding line despite the applied treatment, whereas CLS-1 was in itself more drought tolerant. The line CLS-8 was also found to be drought tolerant per se, most likely due to its overall smaller size, which resulted in reduced total transpiration. CLS-9 had higher yields under drought due to its inherently higher yield potential, albeit this effect was still less pronounced when compared to CLS-2. Finally, CLS-6 performed better than cv Regalona, despite being more sensitive to drought (Table 2).

### 2.2. Thermal Index for Plant Responses to Drought and Irrigation

Thermal index 1 (TI1, °C) was obtained at 46 and 47 days after sowing (DAS), i.e., at the visible inflorescence stage for all plots in both treatments. A TI1 close to zero means cool leaves corresponding to high transpiration, whereas more negative values indicate warmer leaves, suggesting stomatal closure. We found a significant effect of treatment and measurement day, as well as their interaction (*p* < 0.001), but no significant effect of genotype. The measurement day effect originated from the timing of irrigation. At 46 DAS, plants had not been irrigated for almost three days and loss of turgor in leaves was observed in both the FI and RI treatments. All plots were irrigated at noon of 46 DAS, but in the afternoon, TI1 was still very negative in both treatments, indicating that the leaves had not fully recovered and transpiration was still low (Table 3). At 47 DAS, 24 h after irrigation, all values became less negative, suggesting recovery from acute drought, although the treatment effect was noticeable with values closer to zero for FI plants. This was most pronounced for CLS-2, CLS-9 and cv Regalona, and may indicate higher transpiration rates and/or reduced stomatal closure under FI conditions compared to the other lines. For CLS-4 and CLS-8, TI1 values under FI conditions were still more negative, potentially indicating faster stomatal closure and reduced transpiration under high vapor pressure deficit conditions in the afternoon. On the other hand, lines CLS-3 and CLS-4 had the least negative TI1 under RI conditions at 47 DAS, suggesting that transpiration was reduced less, compared to the other lines and cv Regalona.

### 2.3. Hyperspectral Indices as Proxies for Plant Trait Measurements

Hyperspectral imaging data acquired at the visible inflorescence stage were processed to obtain vegetation indices. These included published vegetation indices (VIs, Table S1, [42,43,44,45,46,47,48,49,50,51,52,53,54,55,56,57,58,59,60,61,62,63,64]), calculated based on wavelengths selected for other species and mostly obtained by remote sensing. In addition, we compared the relative reflectance of quinoa across the complete spectral region (File S1).

**Figure 3:**
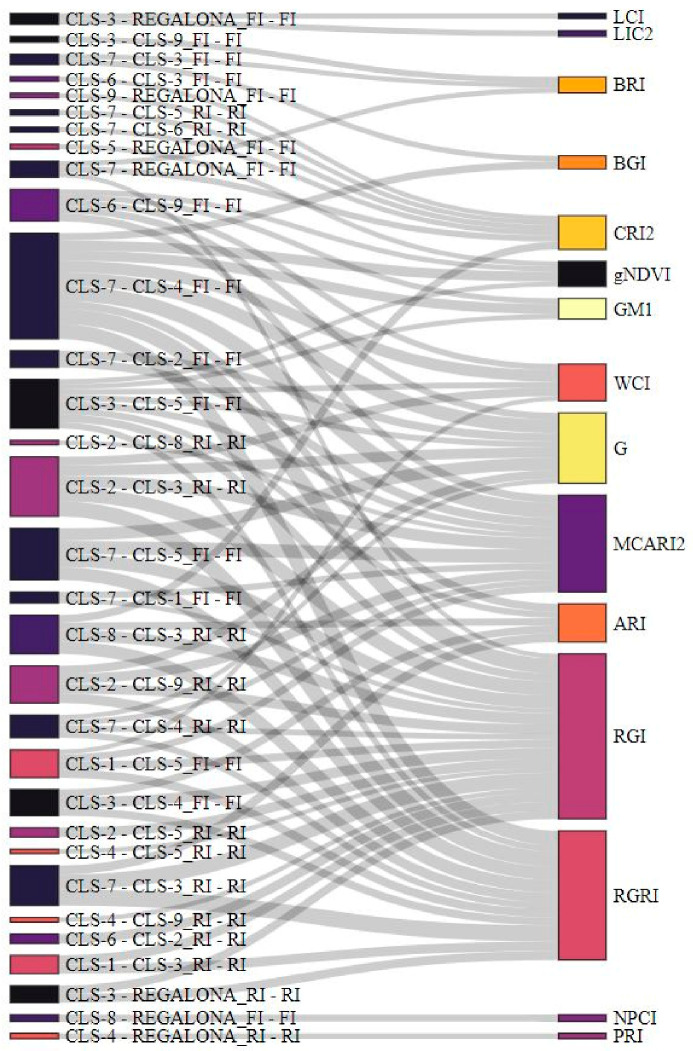
Quinoa genotype pairwise contrasts for the full irrigation (FI) and reduced irrigation (RI) treatments and vegetation indices (VIs) with consistently high effect sizes (larger than one standard deviation) across the three repetitions. The size of the nodes’ rectangle is proportional to the number of connected VIs per genotype contrast (left) or the number of genotype contrasts per VI (right). The online figure is interactive and highlights interactions when pointing the cursor to genotype contrasts and VIs.

We performed a three-way ANOVA to find out whether VIs could detect differences between genotypes and treatments across repetitions. All main effects (genotype, treatment and repetition) were significant, as well as all interactions. We therefore investigated the effect sizes of the main effects and their interactions (Figure S4). Effect sizes were the smallest for treatment in all VIs, which was further confirmed by the results of the two-way ANOVA per repetition. Either genotype or repetition, and their interaction, had the largest effect size depending on the VI. All NDVI-related VIs had very small effect sizes, whereas the RGRI and MCARI2 of the same group of structure-related VIs showed the overall largest effect sizes, together with the RGI and G. Both calculations include wavelengths in the red and green region of the spectrum. RGI and G were followed in effect size by the WCI (red, green, NIR), CRI2 (blue-green, red-edge) and BRI (blue, green). We further analyzed the effect sizes for pairwise contrasts between genotypes within each treatment. Figure 3 shows the VIs for which an effect size larger than one standard deviation across the three repetitions was found. The VIs MCARI2, RGI, RGRI and G showed the largest effect sizes and were associated with a larger number of contrasts between genotypes in both treatments. CLS-7 differed from six other genotypes in the FI treatment, followed by CLS-3 and cv Regalona (five genotypes), CLS-5 (four genotypes), and CLS-9 (three genotypes). In the RI treatment, CLS-2 and CLS-4 appeared at the forefront, with large effect size differences with five and four other genotypes, respectively. CLS-7 and CLS-3 still differed from each other and three other genotypes, whereas cv Regalona only differed from two genotypes.

The WBI, which represents the extent of water-sensitive depression between 900 nm and 970 nm, showed consistent differences in mean values across repetitions between FI and RI treatments for CLS-1, CLS-6 and CLS-7. The effect sizes, on the other hand, were small. The WBI was not different between treatments for CLS-2, CLS-5 and CLS-8. For the remaining lines, no consistent treatment effects were detected. For the other water content-related index WCI, consistent differences in mean values between FI and RI were found for CLS-3, CLS-7, CLS-8 and CLS-9, with effect sizes close to two and three standard deviations for CLS-3 and CLS-9 respectively, and one standard deviation for CLS-7 and CLS-8.

### 2.4. Relationship between Agronomical Traits at Harvest, Plant Phenology, and Morphological and Physiological Traits at the Onset of Flowering

Observations on the progress of phenological stages were made throughout the growing season, but differences between CLS lines and cv Regalona were only found in the duration of the seed maturation stages (File S3). At the visible inflorescence stage (extended BBCH 59 [39]), we measured plant morphological and physiological traits by well-established methods, details of which and measurement results are provided in File S4. Traits included plant height, stem diameter, stem and leaf biomass, stem and leaf water potential, leaf relative water content, and the quantum efficiency of photosystem II.

**Figure 4:**
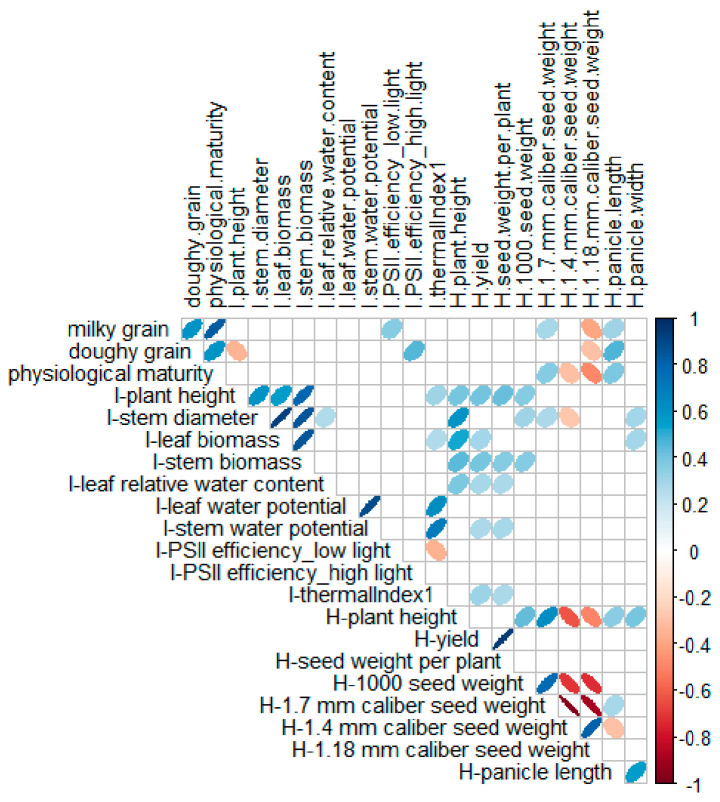
Correlation of phenological, morphological, physiological, and agronomical traits measured across development, at the visible inflorescence stage (traits preceded by ‘I-’), or at harvest (traits preceded by ‘H-’) in nine coastal lowland self-pollinated lines and commercial cv Regalona grown in a field experiment under full irrigation (FI) and reduced irrigation (RI). The color of the ellipses represents the correlation coefficient (R) which can be positive (blue) or negative (red). The slimness of the ellipses represents the coefficient of determination (R^2^). Only significant correlations (*p* < 0.05) are shown.

Morphological traits measured at the visible inflorescence stage showed a significant positive correlation with yield and yield components (Figure 4). Plant height measured at harvest correlated positively with seed size. Among the physiological traits measured at the visible inflorescence stage, the leaf relative water content and stem water potential showed a positive correlation with yield (Figure 4).

**Figure 5:**
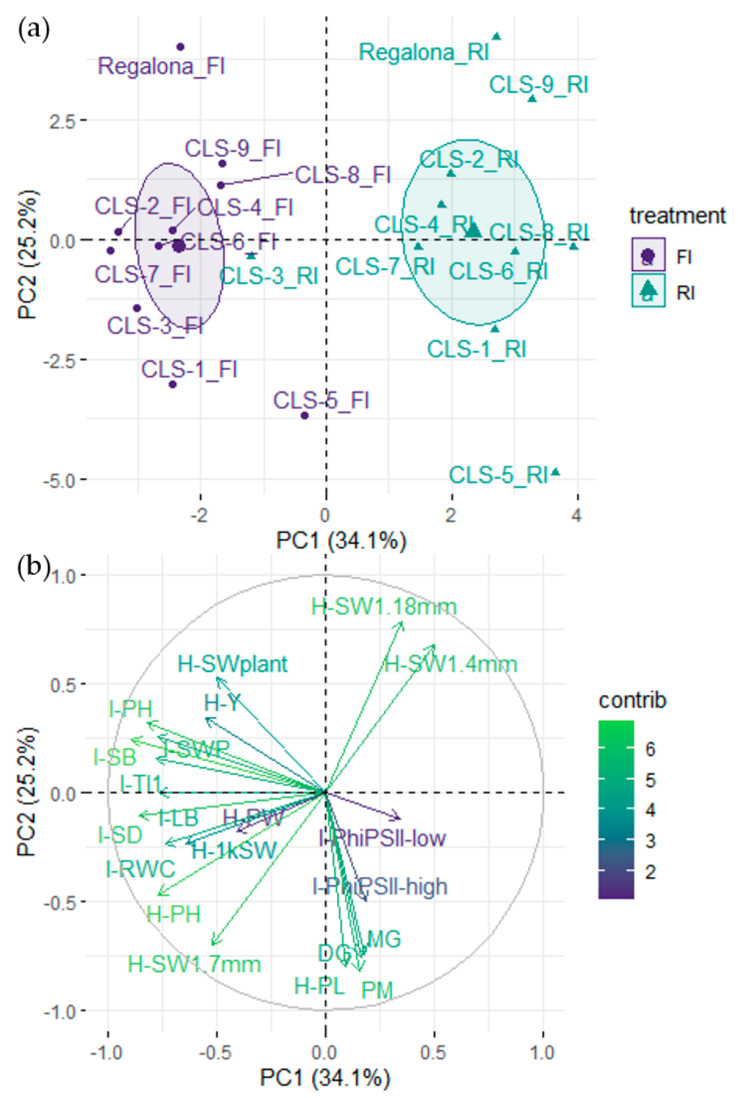
Principal component analysis of agronomical, morphological and physiological traits measured in nine coastal lowland self-pollinated lines (CLS) and commercial cv Regalona grown in a field experiment under full irrigation (FI) and reduced irrigation (RI). (**a**) Distribution of CLS lines and cv Regalona under FI and RI along principal components 1 and 2 (PC1 and PC2); (**b**) Contributions of traits to PC1 and PC2. Phenological traits included in the analysis were: days to milky grain stage (MG), days to doughy grain stage (DG), and days to physiological maturity (PM). Traits measured at the visible inflorescence stage included in the analysis were plant height (I-PH), stem diameter (I-SD), stem biomass (I-SB), leaf biomass (I-LB), leaf relative water content (I-RWC), stem water potential (I-SWP), thermal index 1 (I-TI1), and quantum efficiency of photosystem II in low-light (I-PhiPSII-low) and high-light (I-PhiPSII-high) conditions. Traits measured at harvest included in the analysis were plant height (H-PH), yield (H-Y), seed weight per plant (H-SWplant), 1000 seed weight (H-1kSW), 1.7 mm caliber seed weight (H-SW 1.7 mm), 1.4 mm caliber seed weight (H-SW 1.4 mm), 1.18 mm caliber seed weight (H-SW 1.18 mm), panicle length (H-PL) and panicle width (H-PW).

A principal component analysis (PCA) across treatments explained 59.3% of the variability and separated the treatments along principal component 1 (PC1, 34.1%), with the exception of CLS-3 (Figure 5a). The traits that most contributed to PC1 consisted of morphological traits measured at the visible inflorescence stage, including stem and leaf biomass, stem diameter and plant height (Figure 5b). The latter observation explains why CLS-3-RI did not separate well from CLS-3-FI, because for this line, we did not detect a reduction in shoot biomass under RI conditions at the visible inflorescence stage (File S4). Stem water potential, leaf water content and thermal index 1, measured at the visible inflorescence stage, also contributed to PC1. Treatments were further separated by yield, the 1000 seed weight and plant height at harvest. Principle component 2 (PC2), which explained a further 25.2% of the variability, distinguished CLS lines from cv Regalona, with CLS-1 and CLS-5 being the most distant from cv Regalona as well as the other CLS lines under FI conditions (Figure 5a). Under RI conditions, CLS-5 remained the most distant, whereas CLS-9 was similar to cv Regalona. The traits that most contributed to PC2 were the timing of the milky and doughy grain stages, physiological maturity of the grains, seed size, panicle length, seed weight per plant and the efficiency of PSII under high-light conditions (Figure 5b). According to the PCA, plant height, stem biomass, stem water potential and thermal index 1, all measured at the visible inflorescence stage, were most closely related to yield, whereas panicle length and efficiency of PSII were associated with seed maturation stages. Plant height at harvest was related to seed size (Figure 5b). We did not find any similarity between the classification of the lines according to the DTI and YTI and the position of the lines in the PCA plot.

In a PCA of vegetation indices in combination with agronomical, morphological and physiological traits, PC1, PC2 and PC3 explained 88% of variability (Figure S5). A majority of traits contributing the most to the PC1-3 consisted of VIs developed as proxies for chlorophyll content. Only one water-related VI was in the top ten traits of PC1. The top-scoring VIs corresponded to the VIs for which a consistently large effect size across the three repetitions was found as well as a large number of contrasts between genotypes (Figure 3). For CLS-3 and CLS-7, treatments were most separated along PC1 and PC2 (Figure S5), whereas CLS-4 and CLS-5 (FI and RI), and CLS-6, CLS-9 and CLS-3 (RI) grouped together along PC1. For lines CLS-1 and CLS-4 and cv Regalona, treatments did not separate along PC1 or PC2. No consistent correspondence between DTI and YTI and clustering of lines was detected based on VIs. Also, no significant correlation with yield was found for any of the VIs, and only NPCI and SIPI showed a weakly significant correlation with 1000 seed weight (Table S2). The WBI correlated weakly but significantly with the 1.7 mm and 1.18 mm caliber seed weights. A majority of significant correlations were observed for morphological and physiological traits, with VIs including blue and red wavelengths correlating with morphological traits and stem and leaf water potential, and those calculated using green wavelengths correlating with leaf relative water content (Table S2). The strongest correlations overall were detected for MCARI and PSII efficiency, and PRI and stem and leaf water potential.

## 3. Discussion

Tolerance of quinoa to abiotic stresses such drought, salinity, low soil fertility and frost has been well documented, making it a target crop for addressing future food security in the context of a climate crisis [13,19,20,31,65]. Studies have recorded significant yield deficits, especially under low soil water availability and high vapor pressure deficit, high temperatures and nitrogen deficiency [15,66,67,68,69,70]. Inability to attain the full yield potential has been attributed to sink limitations, while higher yields could be obtained in quinoa if reproductive partitioning is increased [71]. Chilean quinoa varieties from the coastal lowland ecotype have become the basis of most breeding programs aimed at production in temperate environments such as northern Europe, due to their photoperiod insensitivity [32,34]. A high-quality reference genome assembly [72] based on the sequencing of the Chilean coastal quinoa accession PI 614886 (also known as NSL 106399 and QQ74) will contribute significantly to deeper understanding of the genetic attributes of quinoa and to further improvement of future breeding programs. The CLS lines grown in this study all belonged to the coastal lowland ecotype and were selected based on their yield potential across different geographical locations and seasons, architectural traits, and broad adaptability to adverse environmental conditions.

In quinoa, the flowering and grain-filling stages are considered the most critical for yield determination and the most sensitive to stress, including drought and high temperatures [18,68,70,73,74]. Indeterminate grain development in a complex panicle structure coupled with uneven grain filling lies at the basis of this sensitivity [67]. Stress induced by reduced irrigation was well established at the flowering and seed filling stages in our field trial and resulted in significant reductions in yield for the majority of CLS lines, but not in cv Regalona. Lower individual seed weight and a shift in seed size distribution from larger to smaller seeds were also observed. Individual seed weight and seed size were consistently among the smallest in cv Regalona, but total seed number may have compensated for this, as yield in cv Regalona was, on average, similar to the CLS lines. Grain number was the major component in grain yield determination, while grain weight showed a weak to strongly negative association with grain number across a multi-environmental evaluation for grain yield and its physiological determinants [75]. Nevertheless, seed size is an important commercial trait in quinoa [76] and breeding potential is considered to exist for both seed number and seed size [29,30,77,78]. The CLS lines showed potential in field performance for both yield and seed size, even though coastal lowland lines are characterized by a seed caliber under 2 mm. Indeed, fully irrigated (FI) plants yielded 3.24 t ha^−1^ on average, which decreased to 2.45 t ha^−1^ in reduced irrigation (RI) conditions. Nevertheless, in southern Mediterranean conditions the total seed yield ranged from 0.70 t ha^−1^ to 3.25 t ha^−1^, even across seasons [79]. Considering recent reports from arid regions, quinoa Q26 produced the highest seed yield in Bastam, Iran (1.29 t ha^−1^ on average), which was not significantly different from Q29 (1.24 t ha^−1^), while in Damghan, the highest seed yield was achieved in Q26 (1.19 t ha^−1^) [80]. Another report from an arid growing site in China recorded the 1000 seed weight differences for two seasons (2.12–2.03 g) when soil matric potential decreased (−55 kPa SMP), which was significantly lower than under −15 kPa SMP (2.28–2.21 g), well below the averages of 1000 seed weight determined in this study [81].

Yield was maintained under RI in CLS-1 and CLS-2 as well as in cv Regalona, and was relatively sustained in CLS-9 despite the reduced water supply. Higher yields were recorded for both CLS-2 and CLS-9 compared to cv Regalona, which was similar in yield to CLS-1. High yields were achieved using different strategies for leaf relative water content, stem water potential, biomass and seed number and size. This response was similar to observations in the cvs Illpa and Rainbow, which used different strategies in the face of water deficit stress to prevent decreases in grain yield and quality under drought conditions [25]. At the visible inflorescence stage, CLS-2 had one of the largest recorded leaf biomasses, but also suffered the largest reduction in shoot biomass in response to the reduced water supply. High yields in CLS-2 were achieved with medium to large seeds on a short, compact panicle. In cv Regalona, a larger proportion of smaller caliber seeds was produced on a relatively short panicle. CLS-1 produced a higher proportion of large seeds on a long panicle, but overall seed size was lessened by reduced irrigation. Notably, CLS-1 seeds took a longer time to reach the doughy stage of seed maturation, whereas the overall shortest seed maturation time was recorded for the relatively small seeds of cv Regalona. Variation in grain weight was found to be strongly correlated with the rate of grain filling, and weakly or not associated with grain-filling duration [67,73], but this was not observed in our trial. CLS-9 was high yielding, mainly in terms of seed number, as it had a larger proportion of small seeds on a short panicle, like the phenotypic observations of cv Regalona. Notably, the TI1 suggested higher rates of transpiration under FI conditions for CLS-2, CLS-9 and cv Regalona.

CLS-7 had one of the highest proportions of large seeds recorded for both FI and RI treatments, although these were less numerous overall and were produced on a small panicle. Yield was, on average, severely reduced in CLS-5. Plants were short compared to other lines at the visible inflorescence stage, which was partially compensated for by a large panicle at harvest, although a lower proportion of small seeds compared to other lines was observed. CLS-8 already showed reduced stem and leaf biomasses under conditions of RI at the visible inflorescence stage. These effects were more pronounced compared to corresponding observation in cv Regalona. At harvest, CLS-8 plant height was low compared to other CLS lines, despite having a large panicle in both FI and RI treatments. On average, the lowest 1000 seed weight was recorded for CLS-8 and CLS-6, but overall seed yield was not consistently reduced in plots compared to FI. CLS-6 produced a fair yield in terms of seed numbers, but with a higher proportion of small (less than 1.7 mm caliber) seeds. CLS-8 was considered drought tolerant per se, which, according to the TI1, may have been related to very sensitive stomatal closure. Finally, CLS-3 exhibited tall plants both at the visible inflorescence stage and at harvest. While vegetative biomass was not reduced, inflorescences were smaller under RI and yield was reduced mainly due to a reduction in seed number, as CLS-3 produced the overall highest proportion of large seeds and had the heaviest 1000 seed weight. For the CLS lines, plant height at harvest was not correlated with yield, whereas lines with the tallest plants under RI, CLS-3, CLS-7, and CLS-1 and CLS-5 produced the highest proportion of large seeds under both FI and RI conditions (R^2^ of 39%). Lines that showed the highest average yields, CLS-6 and CLS-9, generally had a higher proportion of smaller seeds, with the exception of CLS-2, which produced medium to large seeds.

Correlations between traits measured at the visible inflorescence stage and those measured at harvest were weak overall, most likely due to the small number of measurements per line taken at the visible inflorescence stage and the environmental conditions between flowering and harvest that affect plant physiology and, consequently, seed maturation. Nevertheless, these correlations may give indications about the influence of reduced irrigation from the branching to the visible inflorescence stage on the final yield and seed weight, and about the potential to predict agronomical traits from morphological and physiological traits measured earlier in the growing season. Between 15 and 35% of the variation in plant height at harvest and 5 to 16% of the variation in yield could be explained by shoot biomass measured at the visible inflorescence stage. The PCA analysis also confirmed a relationship between shoot morphology traits at the onset of flowering and yield. Among the physiological traits, leaf relative water content, stem water potential and the TI1 showed significant positive correlations with yield, indicating that these traits contribute to effects of reduced irrigation on yield. Moreover, a significant correlation was found between the TI1 and stem and leaf water potential (R^2^ of 0.49 and 0.39 respectively), supporting the value of thermal infrared imaging for detecting differences in water use behavior among genotypes.

Plant drought response mechanisms have been reported in quinoa and include reduced growth [23,69], stomatal closure associated with abscisic acid and hydraulic signaling [22,25,82,83,84], peroxisome abundance as a cellular sensor [68], the accumulation of osmoprotectants, antioxidant defense and membrane stabilization [19,20,26,27], and elevated recovery capacities of PSII and PSI photochemical activities after re-watering [85]. Understanding how the physiological mechanisms employed by quinoa in response to drought as well as specific strategies implemented by different genotypes influence the final yield is crucial for both crop management and breeding. Better comprehension of genotype by environment (G × E) interactions and the optimization of irrigation for a specific crop’s needs or to address an irrigation deficit [19,25,29,67] will lead to improved crop management (M) and higher-yielding harvests, improving G × E × M interactions. Nevertheless, we were unable to make clear distinctions between quinoa lines employing profligate or conservative water-use strategies [86] using the data collected in this trial via more traditional methods, as the frequency and number of measurements were relatively low. Canopy temperature and vegetation indices collected using handheld, ground-based or remote sensors could distinguish irrigated from non-irrigated treatments in a study by Sankaran et al. [38]. A significant relationship between water availability and canopy temperature was also detected for a selection of quinoa genotypes grown under different water regimes in the Brazilian Cerrado region [70]. We therefore included thermal infrared and hyperspectral imaging to determine whether these systems could deliver proxies for water use, photosynthetic activity or yield, or otherwise reveal differences between lines that may or may not have been observed using standard methods [36,87,88,89,90,91,92].

Both imaging techniques were fast and delivered data with a high temporal resolution and an increased frequency for a potentially better comparison of physiological responses between genotypes and treatments. The Huasco experimental center located in the southern Atacama Desert provided the ideal environmental conditions for this field trial. This was especially apparent for the measuring period at the visible inflorescence stage, where stable conditions coupled with high light intensity and clear skies throughout the day were ideal for applying imaging techniques. In addition, all measuring days had similar diurnal temperature and vapor pressure deficit profiles. The main factor that influenced plants differently was included in the trial itself as the treatment, namely soil water content. The observed responses to acute water deficit and irrigation (re-watering) in the measurement of stem and leaf water potential were also seen in the thermal and vegetation indices as the measurement day had a significant effect.

Further valuable information on the sensitivity of physiological processes to soil water content in the different CLS lines could have been obtained by means of diurnal imaging. With regard to the methodology to obtain the thermal indices, we were fortunate to observe wet leaves (dew) until quite late in the morning, when other environmental conditions (air temperature, radiation, VPD) were already at or close to the maximum values for the day. Covering leaves with petroleum jelly to obtain dry reference temperatures is not ideal, albeit a commonly used method. A more pragmatic approach would be to image a black reference target that adapts quickly to the prevailing environmental conditions, such as a thin sheet of aluminum [93]. An even, black reference surface can be reliably selected with automated image processing procedures. Data obtained from diurnal measurements before and after irrigation, as well as visible loss of turgor, may also help in setting thresholds on indices similar to the upper and lower baselines used in thermal imaging for irrigation scheduling [94].

In both the vegetation indices and the analysis of selected wavelengths, differences between lines were larger than treatment effects. The latter may have been influenced by the measurement days, as these included times of acute water deficit stress and recovery from stress after irrigation. Overall, the mean reflection in the green wavelengths was substantially decreased in RI conditions for most lines, except CLS-2, CLS-4 and CLS-7. Lower green reflection has been previously described in maize under conditions of drought stress [55]. This means that leaves appear darker green, which may be related to a higher concentration of chlorophyll in reduced mesophyll cell volumes and an accumulation of protective pigments [58]. Higher mean NIR reflection has been attributed to a decreased leaf water content and leaf thickness [55,95], and was observed in CLS-5, CLS-6 and CLS-7 in the RI treatment. The blue and green regions of the spectrum contributed the most in distinguishing genotypes, whereas the effect of the measurement day was most clearly observed in the red, red-edge and NIR region of the spectrum (File S1). The time interval since the last irrigation event may therefore have influenced these wavelength regions. Vegetation indices that consist of these wavelengths were the most effective in showing the measurement day effect or distinguishing lines. They included MCARI2, RGI, RGRI and G, consisting of green and red wavelengths, followed by WCI (green, red and NIR), CRI2 (blue, green and red-edge) and BRI (blue and green). CLS-7 and CLS-3 differed from many of the other lines in the FI treatment, whereas they were preceded by CLS-2 and CLS-4 in the RI treatment. The reasons that CLS-3 and CLS-7 strongly differ from each other, the other CLS lines and cv Regalona, based on spectral reflectance, are currently unknown. Nevertheless, both produced the highest proportion of large seeds and the highest 1000 seed weight.

The two water-related VIs could consistently detect differences between FI and RI plots. For the WBI, this was the case in CLS-1, CLS-6 and CLS-7, and for WCI in CLS-3, CLS-7, CLS-8 and CLS-9. No treatment effect was found for the WBI in CLS-2, CLS-5 and CLS-8. The WBI is based on the extent of the water-sensitive depression between 900 nm and 970 nm in the NIR [59], whereas the WCI includes wavelengths in the green, red and NIR regions [55]. In a study by Hinojosa et al. [37] using a handheld multispectral radiometer, the NDVI was suggested as a proxy for yield in quinoa. We could not confirm this result because no consistent relationship was found between the highest yielding CLS lines and their respective NDVI values. We did observe the highest gNDVI under FI conditions for CLS-4, which is known for its dark green leaves and red pigmentation. A similar observation was noted in Hinojosa et al. [37] for a genotype with red shoot coloration. Here, the gNDVI for CLS-4 was high because of a particularly low reflectance in green compared to the other CLS lines and cv Regalona.

No correspondence was found between the classification of lines according to the DTI and YTI and the clustering of lines in the PCA of VIs. In addition, no significant correlation with yield was found for any of the VIs. The majority of significant correlations were observed for morphological and physiological traits, with VIs including blue and red wavelengths correlating with morphological traits and stem and leaf water potential, and those calculated using green wavelengths correlating with leaf relative water content. The PCs mainly consisted of VIs that were originally developed to detect differences in chlorophyll fluorescence and photosynthesis. Potentially, lines CLS-3 and CLS-7 differ from the other lines in these traits. On the other hand, these VIs are also correlated with stem and leaf water potential as the latter has been associated with wavelengths in the red region [55]. We can conclude from this study that hyperspectral imaging has a great potential in estimating traits contributing to yield and in distinguishing genotypes along these traits, rather than providing proxies for yield itself or distinguishing genotypes based on yield.

### Summary and Future Directions

- Significant correlations were detected between morphological and physiological traits measured at the onset of flowering and at harvest.
- Lines CLS-1, CLS-2 and CLS-9 performed best when faced with a 50% reduction in irrigation and performed well in terms of seed traits and plasticity for hyper-arid regions.
- Imaging techniques show good potential for high-throughput phenotyping of quinoa in future studies. Additional data from larger field trials will be needed to improve the quantitative evaluation of quinoa genotypic responses and their relationship to specific traits of interest, including productivity and physiological traits.

## 4. Materials and Methods

### 4.1. Field Trial Setup

Nine novel quinoa genotypes (CLS-1 to CLS-9) and one commercial cultivar (Regalona Baer, referred to in this text as cv Regalona), were grown in the field to investigate responses to full and reduced irrigation. The CLS genotypes were initially selected from the INIA SeedBank Collection based on morphological (i.e., branching type and panicle shape) and yield traits (i.e., total seed weight and seed diameter) observed in the field, and further developed in a breeding program by INIA Chile through a combination of mass selection, self-pollination of individual lines (over the course of at least two seasons) and panicle-furrow selection. The field trial took place at the INIA-Huasco experimental center located in the southern Atacama Desert (Vallenar, Chile, 28°34′ S, 70°47′ W and 469 m.a.s.l.) during the 2019/2020 growing season (sowing on 26 September 2019, harvest on 21 February 2020). This location was particularly well-suited for this trial as the Atacama Desert is known for being one of the driest regions in the world, ensuring that rainfall would not hinder the planned irrigation protocol. The soil corresponded to La Compañía series, a sandy loam textural class of soil composed of sand (75.5%), silt (10.9%) and clay (13.7%), with a low organic matter content (2.0%), an alkaline pH (8.1), slight salinity (2.4 dS m^−1^), and with a basic content of N-P-K (45-21-311 mg kg^−1^ respectively).

**Figure 6:**
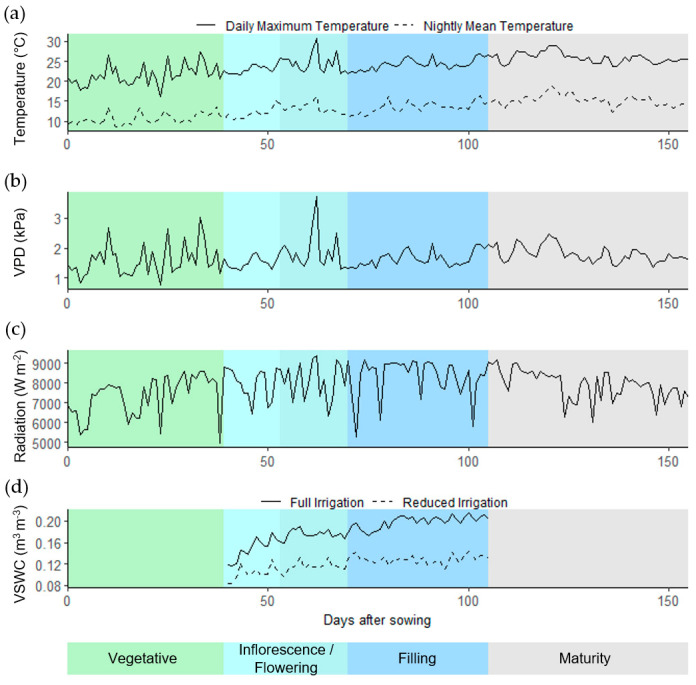
Weather data over the course of the field experiment from 26 September 2019 to 28 February 2020 collected by a meteorological station at the INIA-Huasco experimental center in the southern Atacama Desert (Vallenar, Chile, 28°34′ S, 70°47′ W and 469 m.a.s.l.) 300 m from the field trial and by soil sensors in the field trial; (**a**) daily maximum and night mean temperature; (**b**) daily maximum vapor pressure deficit (VPD); (**c**) daily total radiation; (**d**) daily mean volumetric soil water content (VSWC). The full irrigation and reduced irrigation treatments started at branching (35 days after sowing), but the sensor data were only available from 40 days after sowing. The colors in the figure represent the respective quinoa developmental stages.

The experimental design was split-plot with irrigation treatment as the main plot (between factor, two levels), genotype as the split-plot (within factor, 10 levels) and a blocking factor with six levels (Figure S6). Each plot of 2 by 4 m contained four rows of quinoa with an inter-row spacing of 0.5 m [76]. Seeds were sown at a density of 15 kg ha^−1^ with >85% germination. Two irrigation treatments were tested, with full irrigation (FI) considering the reposition of ET_0_ and the lack of a crop coefficient (K_c_) for quinoa growing in the region. For the reduced irrigation (RI) treatment, we defined a severe reduction of irrigation time (50%) and kept the same schedule as for the full irrigation treatment (every second or third day) by means of pressurized drip lines. The RI treatment started at growth stage 20 (branching, extended BBCH [39]). Fertilizer was provided at sowing (75-120-60 N-P-K) and an additional 75 N was provided at growth stage 12 (second pair of leaves visible, extended BBCH [39]). Plots were regularly inspected for weeds and pests, which were controlled manually, and disease was managed with the application of chlorothalonil (2 L ha^−1^ BRAVO 720, Syngenta) from growth stage 16 (six pairs of leaves visible, extended BBCH [39]). A meteorological station located at the INIA-Huasco experimental station, approximately 300 m from the experimental setup, collected weather data, and soil sensors that monitored volumetric water content and temperature were set up in both treatments (Figure 6). Total monthly precipitation recorded for the field site over the course of the trial was as follows: 0.0 mm from 26 to 30 September, 1.3 mm for the month of October, 0.3 mm for the month of November and 0.2 mm for the month of December. No precipitation was recorded for the months of January and February.

Over the course of the entire growing season, plots were regularly inspected and phenology was determined visually according to the extended BBCH scale by Sosa-Zúñiga et al. [39]. When plants were in the visible inflorescence stage of development (extended BBCH 59 [39]), morphological and physiological traits were measured (described in detail in File S4). The timing corresponded to 46–50 days after sowing (DAS). Morphological traits and leaf relative water content were measured on one day only for all plots of both treatments (47 DAS). Because of practical constraints related to time and the labor-intensive nature of some of the methods, the measurements of the other physiological traits were grouped per block and treatment at 48, 49 and 50 DAS.

### 4.2. Data Collected at Harvest

Plots were harvested during the senescence period once genotypes reached physiological maturity, i.e., when seeds from the main panicle became hard and resistant to pressure, which corresponds to a seed moisture content of about 20%. A 1 m^2^ area consisting of two central rows was manually harvested in each plot. The plant number and height for the harvested area was recorded. Plant yield was determined as the total seed weight in one linear meter of two central rows per plot. Yield data are shown as the productivity in t ha^−1^ at a standard seed moisture content of 20%. Additionally, the total seed weight was divided by the number of plants sampled to obtain the seed weight per plant. The final height was measured for the same plants. Seed metrics included the 1000 seed weight, determined using a seed counting machine (S-JR, DATA Technologies, Kibbutz Tzora, Israel), and the proportional weight of a 10 g seed sample retained in sieves of different mesh openings (1.7, 1.4 and 1.18 mm) after 3 min of agitation at 65 rpm. Finally, panicle metrics were determined for 50 panicles of the central plot rows by imaging complete, intact panicles with a digital RGB camera (Nikon D3100, 16–55 mm lens), and measuring the panicle length and maximum width in the images using ImageJ [96]. A ruler was included in each image for calibration purposes. The drought tolerance index (DTI) and yield tolerance index (YTI) were calculated for each CLS genotype and cv Regalona as described in Ober et al. and del Pozo et al. [40,41].

### 4.3. Thermal Infrared Imaging

Thermal infrared imaging data were acquired using a CAT S60 smartphone equipped with a FLIR Lepton longwave infrared micro thermal camera module (https://www.catphones.com/ (accessed on 5 November 2019)). Images had a resolution of 320 × 480 pixels. Two images per plot were taken to cover the complete plot surface. Images overlapped at the center of the plot, where a fastened piece of crumpled aluminum foil (40 cm^2^) and a leaf covered with petroleum jelly used as a dry reference could be included in both images. Images were acquired at the visible inflorescence stage of development for all plots in both the FI and RI treatments in the afternoon of 46 and 47DAS, whereas they were acquired in the afternoons of 48, 49 and 50 DAS for all plots of blocks 2 and 5, 1 and 4, and 3 and 6, respectively. Moreover, at 47 DAS, all plots were also imaged in the late morning when the leaves were still wet with morning dew. The derived temperature data were used as a wet reference. Images were processed in R using the ‘Thermimage’ package [97]. Settings for air temperature and relative humidity at the time of imaging were obtained from the collected environment data. Emissivity was set to 1 in the conversion of image data for measuring the mean temperature of the crumpled aluminum foil, which represented the reflected temperature. This was then applied together with an emissivity value of 0.96 to obtain the leaf temperature data. An ImageJ macro was used to semi-automatically determine regions of interest (ROIs) in the images and corresponding temperature data for the piece of aluminum foil, the dry reference leaf and patches of sunlit, exposed soil. The temperature data of these ROIs were then excluded from the image to obtain the mean leaf temperature data. Thermal index 1 (TI1) was calculated as follows:

TI1 = dTwet.m − dTm(1)

The dTwet.m is the mean of the difference between the temperature of the wet leaves per plot at 47 DAS and the ambient air temperature at the time of imaging. The dTm is the difference between the mean leaf temperature per plot and the ambient air temperature at the time of imaging [88,89].

### 4.4. Hyperspectral Imaging

Hyperspectral image data were acquired using a Specim IQ (Specim Ltd., Oulu, Finland), a handheld push broom camera system with integrated operating system and controls [98]. The Specim IQ measures reflectance in the visible and near-infrared, i.e., from 400 to 1000 nm, with a spectral resolution (FWHM) of 7 nm, 204 spectral bands, and a spatial resolution of 512 × 512 pixels^2^. The camera was mounted on a tripod at a height that allowed a complete individual plot to be captured in the image. The plots of blocks 1 and 4, and 2 and 5 were imaged at 49 and 48 DAS, respectively, between 15:00 and 16:00. The plots of blocks 3 and 6 were imaged at 49 DAS between 16:00 and 17:00. As blocks represented differences in the date and time of imaging, they were referred to as repetitions with blocks 1 and 4 assigned to repetition 1, blocks 2 and 5 to repetition 2, and blocks 3 and 6 to repetition 3. Plots were always captured in treatment pairs (RI after FI or vice versa). Each dataset contained a white reference tile, which was imaged simultaneously for data calibration. A dark reference, representing sensor noise without incoming light, was recorded automatically before each capture.

Upon image data acquisition, the Specim IQ integrated software allows for the selection of the white reference tile in the image based on its high reflectance values, in addition to automated calibration to obtain relative reflectance data. However, we noted that the white reference tile itself was not selected alone in some images, as other elements with high reflectance were present, such as pieces of crumpled aluminum foil (used for the measurement of stem water potential and thermal imaging). The calibration procedure was therefore redone in R after threshold-based selection of the white reference tile pixels using an ImageJ macro. All other hyperspectral data processing and analysis steps were also executed in R.

Plant pixels were segmented from the background using the Normalized Difference Vegetation Index (NDVI, Table S1) and a threshold level, which also excluded inflorescences and specular reflection. Shaded background and shaded plant parts with low reflectance were removed using a threshold in near-infrared (838 nm) and green (554 nm) wavelengths, respectively. Spectra were smoothed on the pixel level using the Savitzky–Golay smoothing filter [99] with a third order polynomial and a window size of 11 using the R package ‘prospectr’ [100].

A total of 41 published vegetation indices (VIs, Table S1) were calculated. By means of a cluster analysis, genotypes were grouped based on the similarity of VI data within the FI and RI treatment. Agglomerative hierarchical clustering was applied on the scaled VI mean observations for repetitions 1 to 3 using the ‘agnes’ function of the R package ‘cluster’ [101]. The trees were cut at five clusters. Pearson correlation coefficients were calculated to describe the linear relationships between traits measured at the visible inflorescence stage, plant morphology and performance traits measured at harvest, and VIs. In addition, differences in relative reflectance between genotypes and treatments, independent of VIs, were analyzed for a selection of wavelengths and wavelength bands. A selection was used because of the high degree of correlation or collinearity in the relative reflectance of mostly adjacent wavelengths. A Pearson correlation coefficient was calculated between the relative reflectance of all wavelengths. A threshold of 0.8 was then applied to split up the wavelength range in groups of high correlation. One wavelength was selected for further analysis per group. This yielded five wavelengths, 476 nm, 554 nm, 616 nm, 679 nm and 724 nm, in the blue, green, orange, red and red-edge regions of the spectrum, respectively. Furthermore, reflectance in the near-infrared region (NIR) was averaged and included in the selection.

### 4.5. Statistics

Statistics were performed in R (R version 4.0.3 [102]). Outliers were identified by applying the interquartile range method (R package ‘rstatix’ [103]). Data were checked for normality via visual inspection of the QQplot of the residuals of the model and by the Shapiro-Wilk test. Homogeneity of variances was determined by visually inspecting the residuals plot and by Levene’s test.

The model ‘trait ~ treatment × genotype + (1|block)’ was run for harvest data using the ‘lmer’ function in the R package ‘lmerTest’ [104]. Post-hoc pairwise comparisons were performed using estimated marginal means with a 95% confidence interval (R package ‘emmeans’ [105]). For thermal infrared imaging, a three-way ANOVA (TI1 ~ genotype × treatment × measurement day) was run. Significant main effects of treatment and measurement day, and their interaction, were followed by a one-way ANOVA to test the main effect of treatment at all levels of the measurement day, and vice versa.

For hyperspectral imaging, the VIs PSSRa, PSSRb, PSSRc, SR, SRChl, SRChlb and SRChltot, and relative reflectance data at 554 nm were log-transformed, a square root transformation was applied on data at 616 nm, 724 nm and the VI MCARI, and the reciprocal of the data of the VI WCI was used to improve normality before statistical testing could be performed. For VIs and each selected wavelength or wavelength band, a three-way ANOVA (vegetation index or wavelength ~ genotype × treatment × repetition) was run. Assumptions were checked based on the normal QQ plot and the residual plot. Statistical tests for normality or homogeneous variance could not be used because of the large sample size. The latter also affected the outcome of the ANOVA as all main and interaction effects were highly significant, and only very few non-significant contrasts (genotype and treatment) were detected. Effect sizes are therefore reported here. The three-way ANOVA was followed by a two-way ANOVA (vegetation index or wavelength ~ genotype × treatment or repetition). Effect sizes for the independent variable in the ANOVA models were the generalized eta squared, whereas they were calculated as pairwise differences of estimated marginal means, divided by the standard deviation of the population, for pairwise comparisons between genotypes and treatments.

Pearson or Spearman correlation coefficients, depending on the distribution of the trait data, were calculated to describe linear relationships between traits and indices measured at the visible inflorescence stage and agronomical traits measured at harvest (R packages ‘Hmisc’ [106] and ‘corrplot’ [107]). The mean values of traits per plot were used. A principal component analysis (PCA) was performed on the mean values of traits and indices per genotype and treatment using the ‘prcomp’ function in the ‘stats’ package [102]. The ‘factoextra’ package was used for the visualization of the PCA [108].

## Figures and Tables

**Table 1:** One-thousand seed weight and proportional weight of 1.7 mm caliber seeds measured for nine coastal lowland self-pollinated lines (CLS) and commercial cv Regalona in a field experiment under full irrigation (FI) and reduced irrigation (RI). Values are means across treatment ± standard deviation.

Genotype	1000 Seed Weight (g)	1.7 mm Caliber Seed Weight (g) ^1^
CLS-1	3.22 ± 0.25 ab	5.93 ± 1.10 a
CLS-2	3.22 ± 0.19 ab	4.35 ± 0.92 b
CLS-3	3.28 ± 0.17 a	5.83 ± 0.51 a
CLS-4	3.02 ± 0.08 bc	4.23 ± 0.54 bde
CLS-5	2.82 ± 0.26 cde	4.80 ± 1.13 bc
CLS-6	2.68 ± 0.19 de	3.78 ± 0.66 def
CLS-7	3.25 ± 0.10 a	5.67 ± 0.55 ac
CLS-8	2.60 ± 0.26 e	3.35 ± 0.72 ef
CLS-9	2.90 ± 0.40 cd	3.05 ± 1.59 fg
Regalona	2.73 ± 0.16 de	2.35 ± 0.86 g

^1^ Out of a 10 g sample of seeds, the total weight of seeds that are larger than 1.7 mm. *n* = 6. Letters indicate significant differences between lines (*p* < 0.05).

**Table 2:** Drought and yield tolerance indices according to Ober et al. [40] and del Pozo et al. [41] calculated for nine coastal lowland self-pollinated lines (CLS) and commercial cv Regalona grown in a field experiment under full irrigation and reduced irrigation. The drought tolerance index (DTI) is based on the drought intensity index across all genotypes in the experiment (DII, 0.76), whereas the DTI-R is based on the DII of Regalona (0.93) as a reference. The yield tolerance index (YIT) is calculated across all genotypes in the experiment.

Genotype	DTI	DTI-R	YTI
CLS-1	1.41	1.15	0.66
CLS-2	1.32	1.08	1.29
CLS-3	0.96	0.78	0.69
CLS-4	0.78	0.63	0.67
CLS-5	0.60	0.49	0.36
CLS-6	0.88	0.71	1.01
CLS-7	0.90	0.74	0.55
CLS-8	1.02	0.83	0.66
CLS-9	0.98	0.80	1.07
Regalona	1.23	1	0.72

**Table 3:** The thermal index 1 (TI1, °C) calculated based on thermal infrared data for nine coastal lowland self-pollinated lines (CLS) and commercial cv Regalona in a field experiment under full irrigation (FI) and reduced irrigation (RI) conditions. Images were acquired at the visible inflorescence stage on two consecutive measurement days, expressed in days after sowing (DAS). Values are means ± standard deviation.

Genotype	46 DAS		47 DAS	
	FI	RI	FI	RI
CLS-1	−5.55 ± 0.65	−5.39 ± 0.38	−1.10 ± 0.85 †	−2.72 ± 0.61 †
CLS-2	−5.00 ± 1.72	−4.17 ± 0.79	−0.02 ± 1.23 †	−2.68 ± 1.04 *
CLS-3	−5.21 ± 0.74	−3.85 ± 0.78	−0.37 ± 1.20 †	−1.69 ± 0.65 †
CLS-4	−6.13 ± 1.62	−4.38 ± 0.20	−1.70 ± 0.27 †	−1.87 ± 0.88 †
CLS-5	−5.07 ± 1.38	−5.96 ± 1.37	−1.10 ± 1.28 †	−2.27 ± 0.79 †
CLS-6	−5.03 ± 1.42	−4.91 ± 1.96	−0.81 ± 1.12 †	−2.38 ± 1.24
CLS-7	−5.48 ± 0.60	−5.29 ± 0.67	−1.30 ± 1.09 †	−2.57 ± 1.33 †
CLS-8	−5.22 ± 0.88	−5.19 ± 0.54	−2.29 ± 1.06 †	−3.23 ± 0.77 †
CLS-9	−4.24 ± 1.22	−5.06 ± 0.81	−0.12 ± 0.90 †	−3.37 ± 0.64 †*
Regalona	−4.36 ± 1.23	−4.55 ± 1.90	−0.17 ± 0.48 †	−2.75 ± 0.11 *

*n* = 3. * indicates significant differences between treatments per genotype and measurement day (*p* < 0.05). † indicates significant differences between measurement days 46 DAS and 47 DAS within genotype and treatment (*p* < 0.05).

## Data Availability

The data presented in this study are available on request from the corresponding author. The data are not publicly available yet due to variety registration.

## Supplementary Materials

The following are available online at https://www.mdpi.com/article/10.3390/plants11030323/s1, Figure S1: Seed metrics.pdf, Figure S2: Panicle metrics.pdf, Figure S3: Plant height at harvest.pdf, Figure S4: Vegetation indices-effect size.pdf, Figure S5: PCA of vegetation indices.pdf, Figure S6: Field trial plot layout and experimental design.pdf, Table S1: Vegetation indices.pdf, Table S2: Correlations between traits and vegetation indices.pdf, File S1: Analysis of selected wavelengths.pdf, File S2: Selected wavelengths-effect size-pairwise comparisons.xlsx, File S3: Phenology.xlsx, File S4: Morphological and physiological traits at the inflorescence visible stage.pdf.
