# Supplementary material for: Morphological and Physiological Traits Associated with Yield under Reduced Irrigation in Chilean Coastal Lowland Quinoa
Source: Plants (Basel). 2022 Jan 26;11(3):323. doi: 10.3390/plants11030323 (PMC8839172; doi:10.3390/plants11030323)
Supplement: Supplementary file 1 [file plants-11-00323-s001.zip › Figure S1. Seed metrics.pdf]

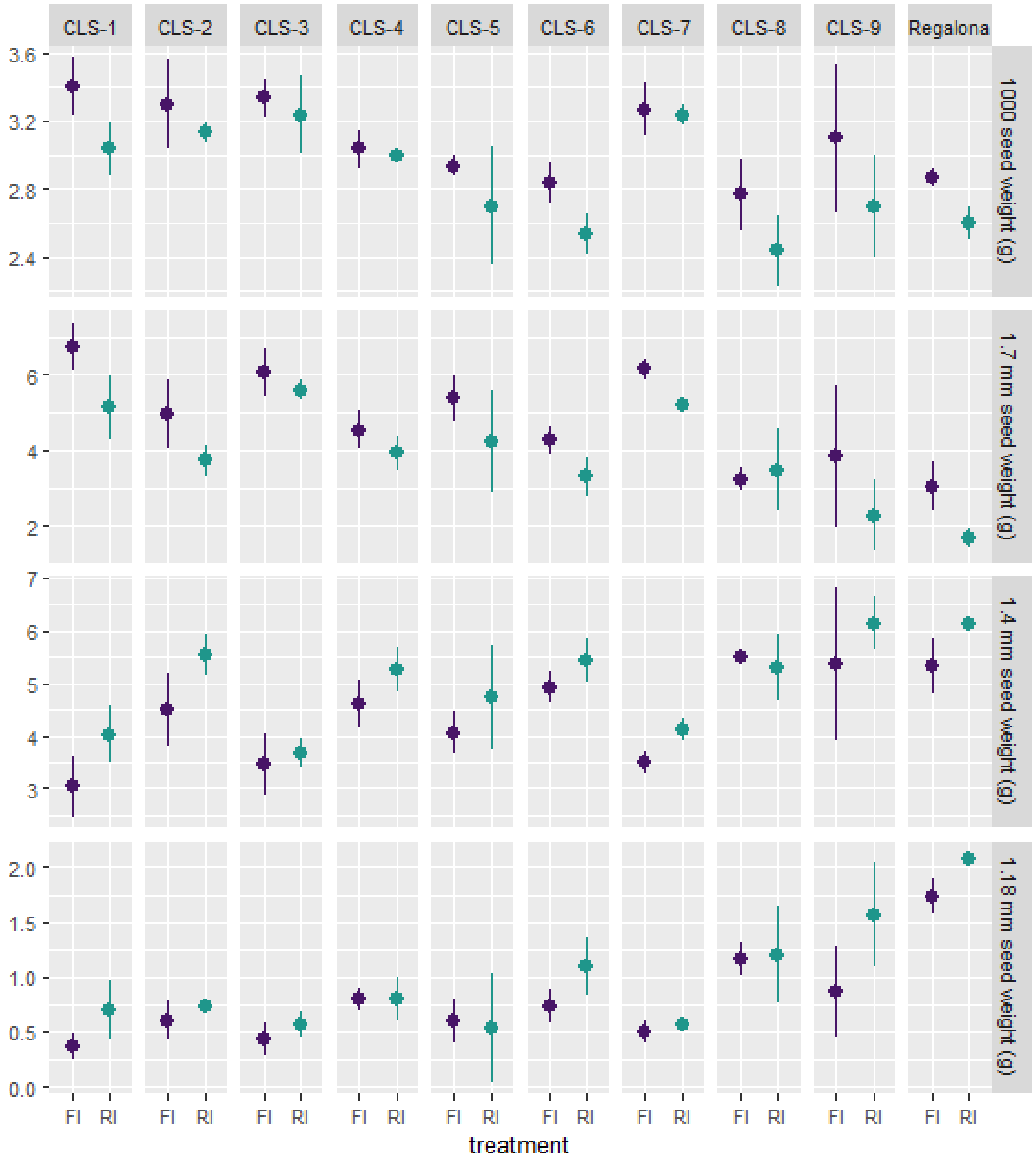

Figure S1. Seed metrics of nine coastal lowland self-pollinated lines (CLS) and commercial cv Regalona in a field experiment under full irrigation (FI, purple) and reduced irrigation (RI, green). Seed metrics include from the top to the bottom panel the 1000 seed weight and the proportional weight of 1.70, 1.40 and 1.18 mm caliber seeds in a 10 g sample of seeds (bottom panel). Data are presented as the mean (dot) and the standard deviation around the mean (vertical bar).
