# Supplementary material for: Morphological and Physiological Traits Associated with Yield under Reduced Irrigation in Chilean Coastal Lowland Quinoa
Source: Plants (Basel). 2022 Jan 26;11(3):323. doi: 10.3390/plants11030323 (PMC8839172; doi:10.3390/plants11030323)
Supplement: Supplementary file 1 [file plants-11-00323-s001.zip › Figure S2. Panicle metrics.pdf]

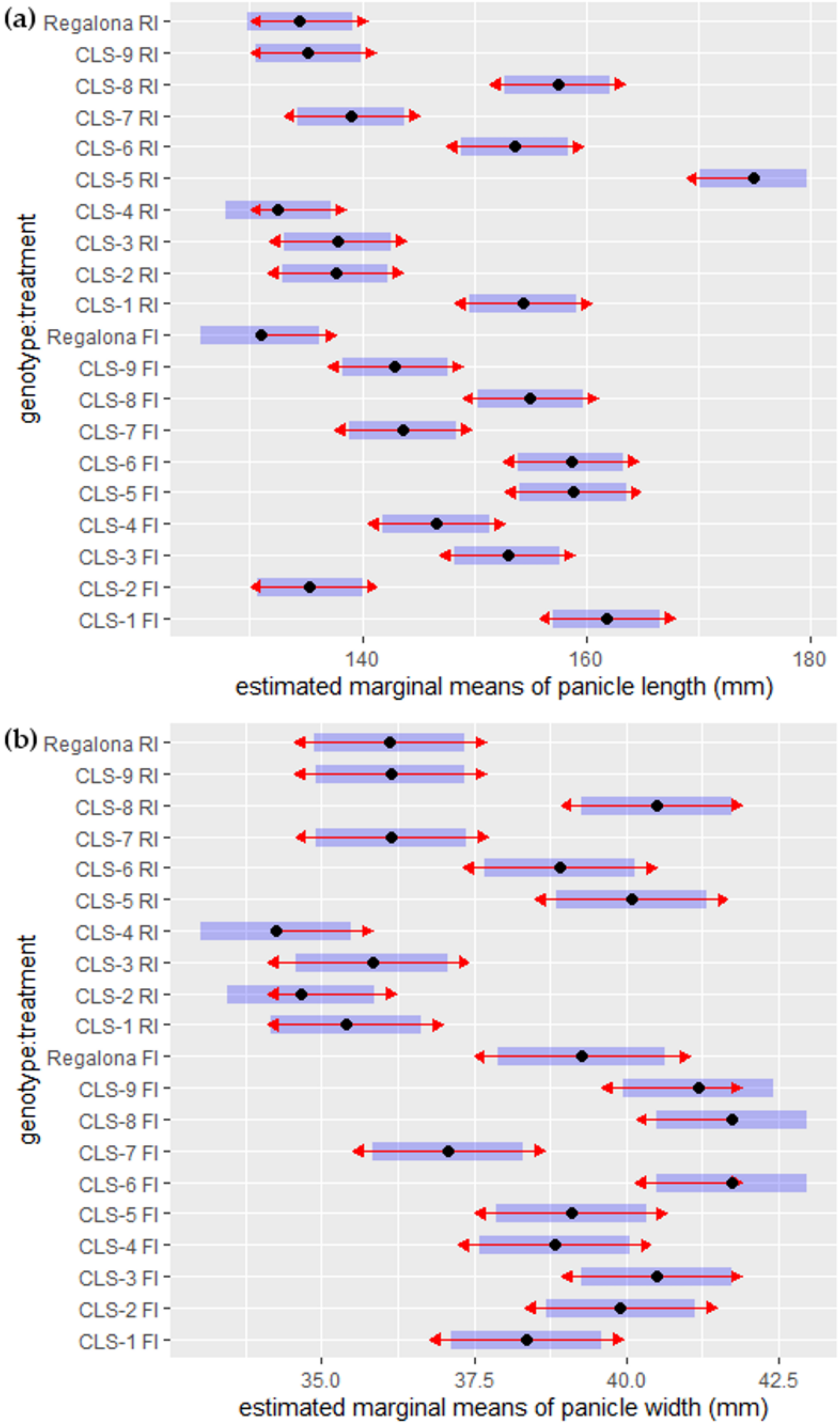

Figure S2. Estimated marginal means of panicle metrics of nine coastal lowland self-pollinated lines (CLS) and commercial cv Regalona grown in a field experiment under full irrigation (FI) and reduced irrigation (RI); (a) Panicle length; (b) Panicle maximum width. The purple bar corresponds to the 95% confidence interval of the estimate (black dot), whereas the degree to which red arrows overlap reflects the significance of the comparison of two estimates.
