# Supplementary material for: Morphological and Physiological Traits Associated with Yield under Reduced Irrigation in Chilean Coastal Lowland Quinoa
Source: Plants (Basel). 2022 Jan 26;11(3):323. doi: 10.3390/plants11030323 (PMC8839172; doi:10.3390/plants11030323)
Supplement: Supplementary file 1 [file plants-11-00323-s001.zip › Figure S4. Vegetation indices-effect size.pdf]

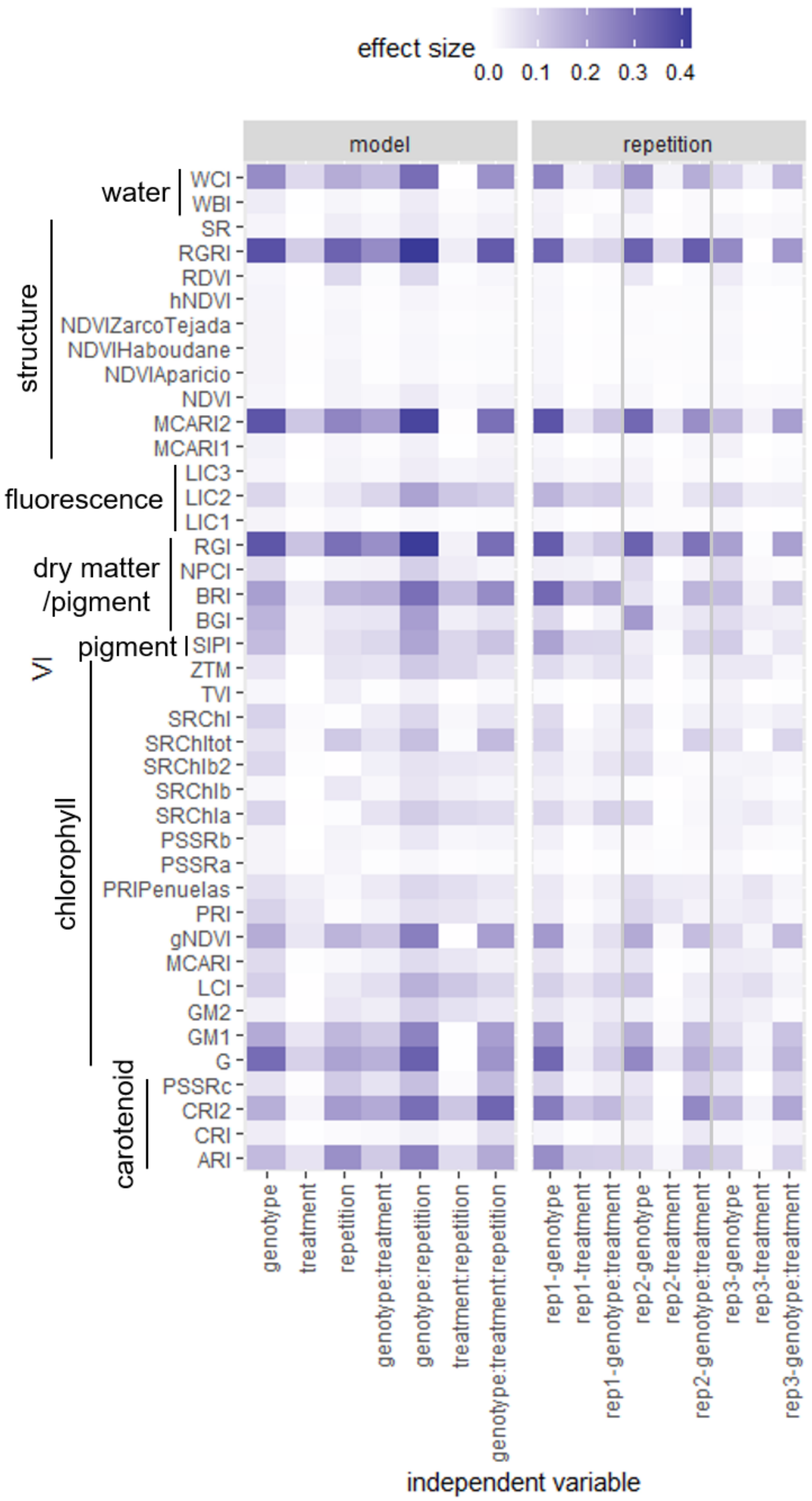

Figure S4. Heat map of effect sizes of independent variables of the three-way ANOVA ('model') and the two-way ANOVA per repetition ('repetition') for vegetation indices (VI) calculated at the inflorescence visible stage for nine coastal lowland self-pollinated lines and commercial cv Regalona grown in a field experiment under two treatments (full and reduced irrigation).
