# Supplementary material for: Morphological and Physiological Traits Associated with Yield under Reduced Irrigation in Chilean Coastal Lowland Quinoa
Source: Plants (Basel). 2022 Jan 26;11(3):323. doi: 10.3390/plants11030323 (PMC8839172; doi:10.3390/plants11030323)
Supplement: Supplementary file 1 [file plants-11-00323-s001.zip › Figure S5. PCA of vegetation indices.pdf]

(a)

(b)

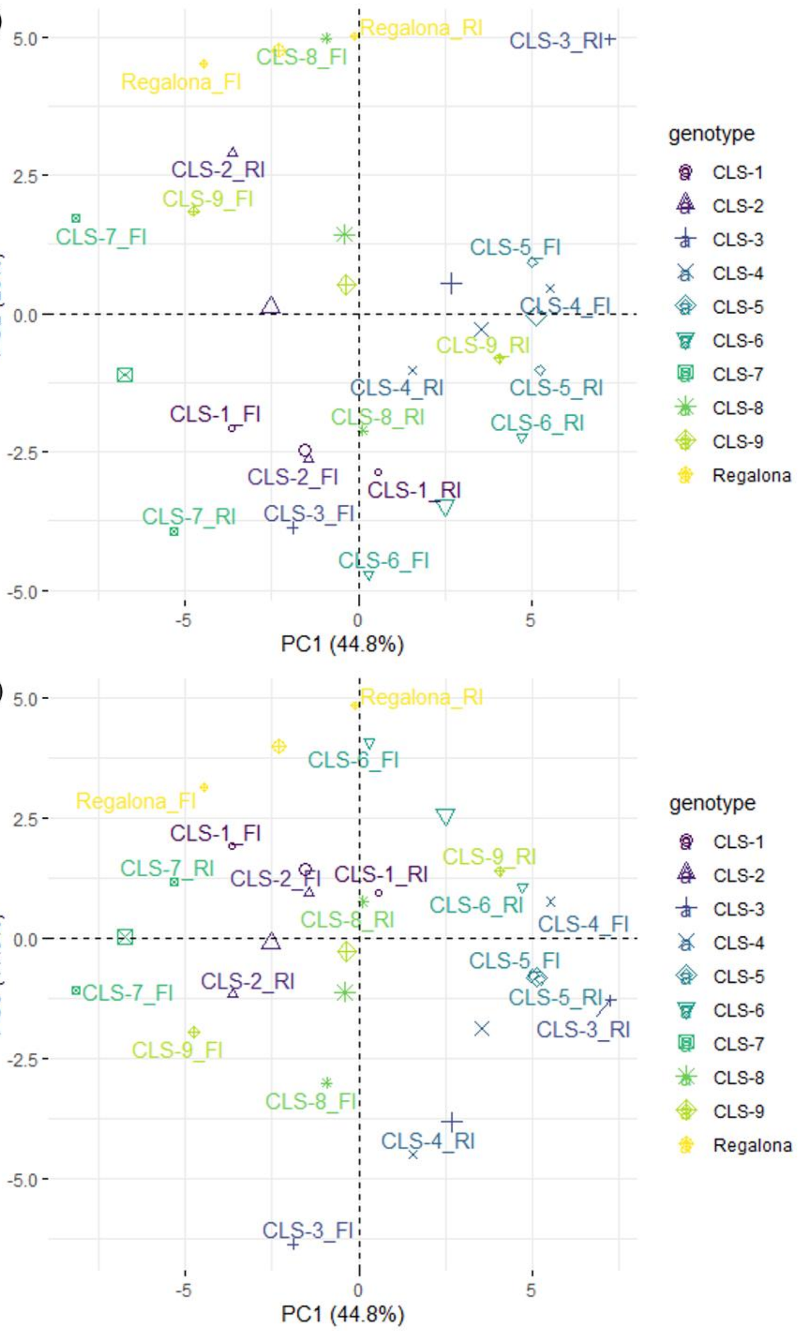

(c)

	+	-
**PC1**	RGI	G
	RGRI	MCARI2
	WCI	GM1
	gNDVI	LIC1
	SRChl	NDVIAparicio
**PC2**	GM2	ZTM
	MCARI	LCI
	CRI	SRChlb
	LIC2	SRChla
	CRI2	PRIPenuelas
**PC3**	SR	BRI
	NDVI	NPCI
	PSSRb	GM1
	SRChltot	SRChlb2
	PSSRc	SRChla

(d)

	+	-
**PC1**	dry matter, pigments	chlorophyll
	structure	structure
	water	chlorophyll
	chlorophyll	fluorescence
	chlorophyll	structure
**PC2**	chlorophyll	chlorophyll
	chlorophyll	chlorophyll
	carotenoids	chlorophyll
	fluorescence	chlorophyll
	carotenoids	chlorophyll
**PC3**	structure	dry matter, pigments
	structure	dry matter, pigments
	chlorophyll	chlorophyll
	chlorophyll	chlorophyll
	carotenoids	chlorophyll

Figure S5. Principal component analysis of vegetation indices (VIs) in combination with agronomical, morphological and physiological traits measured in nine coastal lowland self-pollinated lines (CLS) and commercial cv Regalona grown in a field experiment under full irrigation (FI) and reduced irrigation (RI). (a) Distribution of CLS lines and cv Regalona under FI and RI along principal components 1 and 2 (PC1 and PC2); (b) Distribution of CLS lines and cv Regalona under FI and RI along principal components 1 and 3 (PC1 and PC3); (c) The top five traits (VIs) contributing the most to PC1, PC2 and PC3 in both the positive and negative direction; (d) The sensitivity category of the VIs listed in (c).
