# Supplementary material for: Morphological and Physiological Traits Associated with Yield under Reduced Irrigation in Chilean Coastal Lowland Quinoa
Source: Plants (Basel). 2022 Jan 26;11(3):323. doi: 10.3390/plants11030323 (PMC8839172; doi:10.3390/plants11030323)
Supplement: Supplementary file 1 [file plants-11-00323-s001.zip › Figure S6. Field trial plot layout and experimental design.pdf]

**(a)**

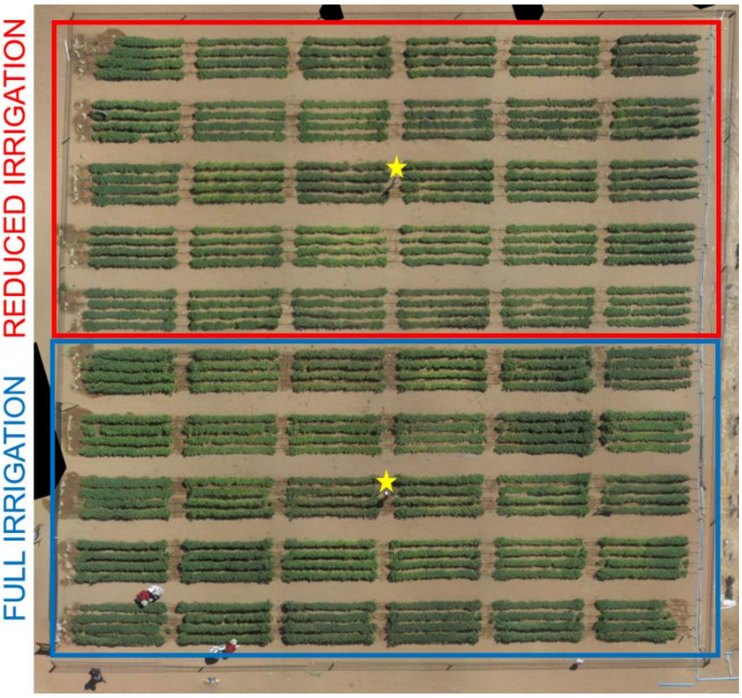


**(b)**

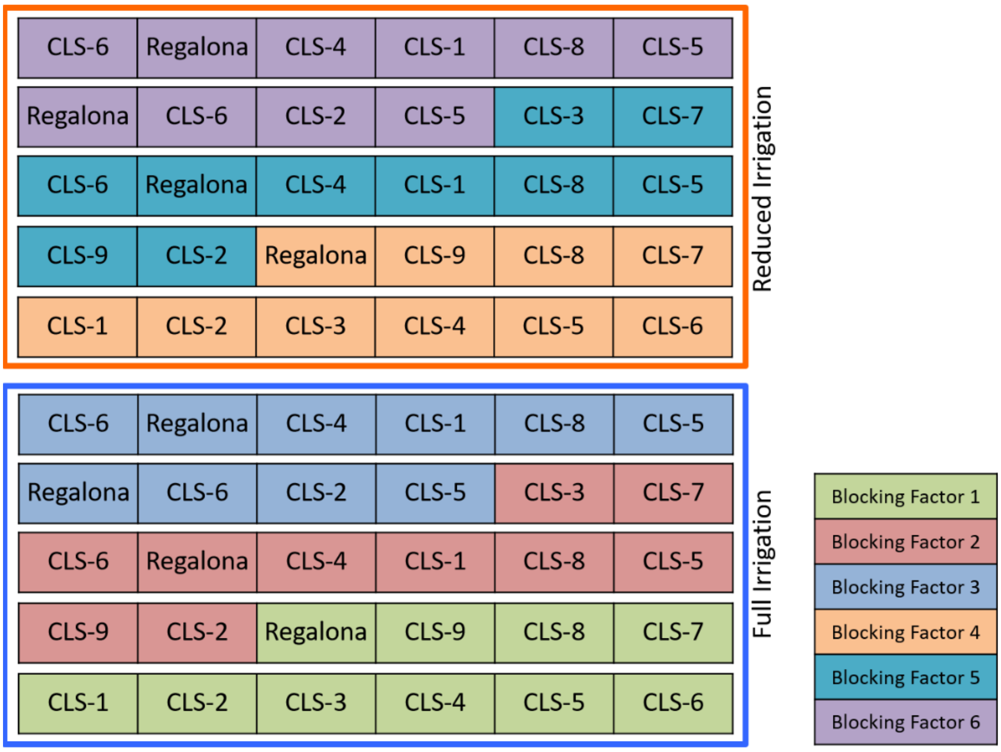


| | | | | | | | | | | |
|---|---|---|---|---|---|---|---|---|---|---|
| Blocking Factor 1 | FI-G1 | FI-G2 | FI-G3 | FI-G4 | FI-G5 | FI-G6 | FI-G7 | FI-G8 | FI-G9 | FI-G10 |
| Blocking Factor 2 | FI-G1 | FI-G2 | FI-G3 | FI-G4 | FI-G5 | FI-G6 | FI-G7 | FI-G8 | FI-G9 | FI-G10 |
| Blocking Factor 3 | FI-G1 | FI-G2 | FI-G3 | FI-G4 | FI-G5 | FI-G6 | FI-G7 | FI-G8 | FI-G9 | FI-G10 |
| Blocking Factor 4 | RI-G1 | RI-G2 | RI-G3 | RI-G4 | RI-G5 | RI-G6 | RI-G7 | RI-G8 | RI-G9 | RI-G10 |
| Blocking Factor 5 | RI-G1 | RI-G2 | RI-G3 | RI-G4 | RI-G5 | RI-G6 | RI-G7 | RI-G8 | RI-G9 | RI-G10 |
| Blocking Factor 6 | RI-G1 | RI-G2 | RI-G3 | RI-G4 | RI-G5 | RI-G6 | RI-G7 | RI-G8 | RI-G9 | RI-G10 |

Between Factor (Main plot) Irrigation treatment with two levels: full irrigation (FI), reduced irrigation (RI)
Within Factor (Split-plot) Genotype (G) with 10 levels

**Figure S6.** Layout of experimental plots (a) for nine coastal lowland self-pollinated lines (CLS) and commercial cv Regalona (REGA) grown in a field experiment under full irrigation (blue frame) and reduced irrigation (red frame), and corresponding experimental design corresponding to a split-plot (b). The yellow stars in (a) indicate the position of field environment measurement stations for soil water content (full and reduced irrigation) and air temperature and relative humidity (full irrigation only). The image in (a) is a composition of RGB image data acquired by a drone at the inflorescence visible stage corresponding to 46 days after sowing.
