# Supplementary material for: Morphological and Physiological Traits Associated with Yield under Reduced Irrigation in Chilean Coastal Lowland Quinoa
Source: Plants (Basel). 2022 Jan 26;11(3):323. doi: 10.3390/plants11030323 (PMC8839172; doi:10.3390/plants11030323)
Supplement: Supplementary file 1 [file plants-11-00323-s001.zip › File S1. Analysis of selected wavelengths.pdf]

Selected wavelengths showed high sensitivity to image acquisition timing

In addition to vegetation indices, we aimed to compare the relative reflectance of quinoa across the complete spectral region. Neighboring wavelengths showed however a high degree of correlation in reflectance, which resulted in collinearity problems. For five auto-correlating wavelength groups, a single central wavelength was selected at 476 nm, 554 nm, 616 nm, 679 nm and 724 nm, which corresponded to the blue, green, orange, red and red-edge region of the spectrum, respectively. The analysis also included the near-infrared region (NIR) for which the average relative reflectance was calculated.
The mean reflectance spectra of the nine coastal lowland self-pollinated (CLS) lines and the commercial cultivar (cv) Regalona under fully irrigated (FI) and reduced irrigation (RI) conditions at the inflorescence visible stage are shown in Figure S1.1, together with the selected wavelengths and the NIR region. The line CLS-4 stood out because of the lower reflectance in the blue, green and orange regions of the spectrum, in particular under FI conditions. This corresponded with its darker green, red pigment-rich leaves. For most lines except for CLS-7, CLS-2 and CLS-4, green reflectance was substantially decreased under RI conditions. In line CLS-5 it decreased to the level of green reflectance of CLS-4, whereas its NIR reflectance markedly increased. The NIR reflectance was the highest for CLS-5, followed by the lines CLS-7 and CLS-6, and may indicate a lower water content [94]. CLS-2 and CLS-7 had the highest green reflection under RI conditions, but CLS-2 showed the lowest reflection in the NIR.

As for the vegetation indices, we ran a three-way ANOVA to detect differences between lines and treatments across repetitions for the selected wavelengths. All main effects (genotype, treatment and repetition) were significant, and also all interactions. We therefore looked at the effect size of main effects and their interactions (Figure S1.2). Effect sizes were very small for treatment for all wavelengths, which was confirmed by the results of the two-way ANOVA per repetition. For genotype and repetition, the effect size depended on the wavelength. In the blue and green regions of the spectrum (476 nm and 554 nm, respectively), the effect sizes were large for genotype and the genotype x repetition interaction, while for the orange region (616 nm), genotype and repetition effect sizes were very similar. Repetition had the largest effect sizes for the red (679 nm), red-edge (724 nm) and NIR region of the spectrum. This means that the plot and/or the time since the last irrigation event may have influenced these wavelength regions. The plots of repetitions 1 and 3 were imaged at 49 days after sowing and less than 24 h after the last irrigation, whereas the plots of repetition 2 were imaged at 48 days after sowing and 2 days after the last irrigation.

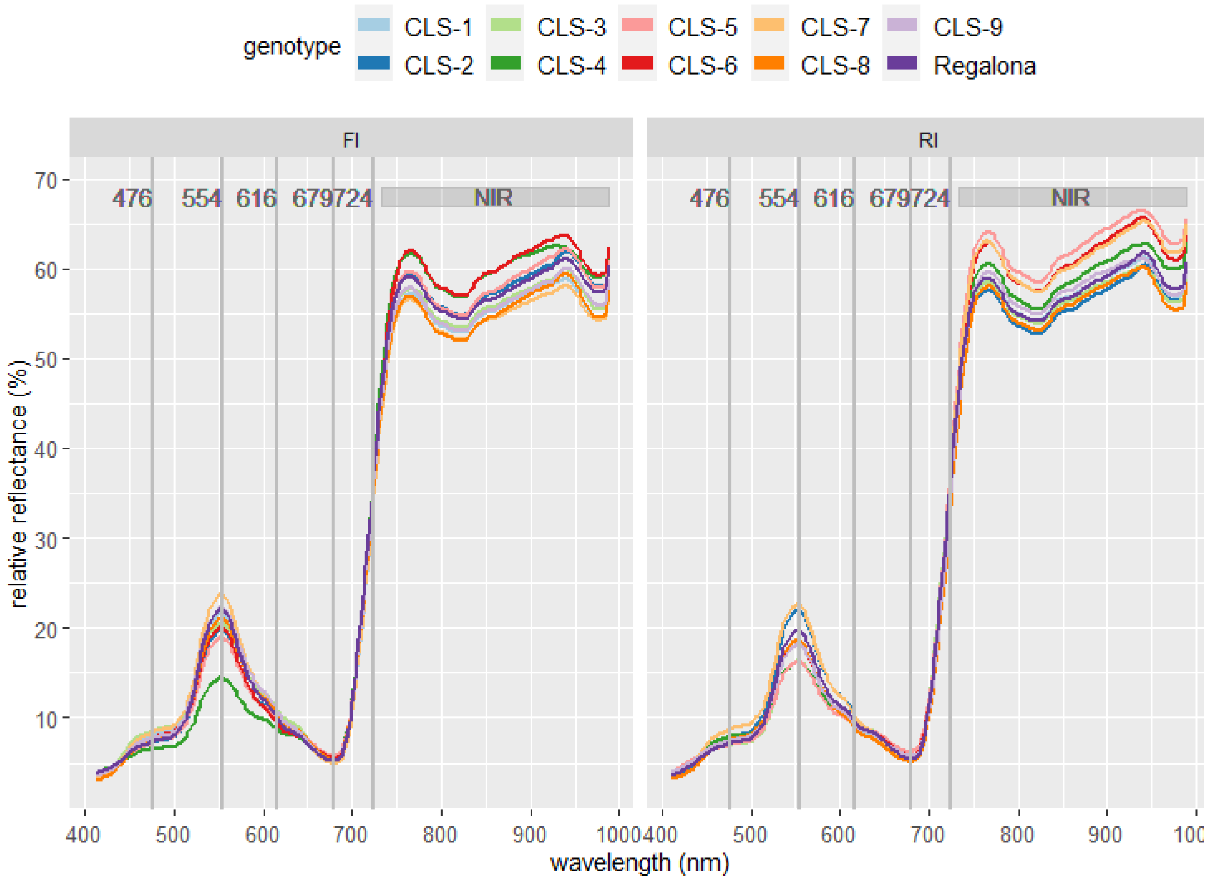

Figure S1.1. Mean relative reflectance spectra at the inflorescence visible stage of nine coastal lowland self-pollinated lines (CLS) and commercial cv Regalona in a field experiment under full irrigation (FI) and reduced irrigation (RI). The five wavelengths and the near-infrared region (NIR) included in the comparison between genotypes and treatments are indicated by vertical lines and a horizontal bar, respectively.

We further analyzed the effect sizes for pairwise contrasts between genotypes within each treatment and across treatments. Overall, effect sizes were smaller than those observed in the vegetation indices. Pairwise contrasts with absolute effect sizes larger than 0.5 standard deviation across the repetitions are reported in Table S1.1. We noticed that the sign of the effect size for repetition 2 was often the opposite of the effect size of repetitions 1 and 3, also in the FI condition, which may indicate an effect of time passed after the last irrigation event and sensitivity to a decreasing soil water content. This would then be larger than a plot effect and may confound treatment effects. It was observed for 476 nm, 554 nm and 616 nm within the FI and RI treatments (Table S1.1), but was also present for the other wavelengths with smaller effect sizes (File S2). Within genotypes, we found no consistent effect sizes for treatment across the repetitions. For the red region of the spectrum (679 nm), only small effect sizes (<0.5 standard deviations) were obtained, and those for the red-edge (724 nm) and NIR were also small in general. Within the FI treatment, lines CLS-7 and CLS-9 showed the highest number of 'largest effect size' contrasts with other lines, whereas within the RI treatment, the line CLS-2 stood out.

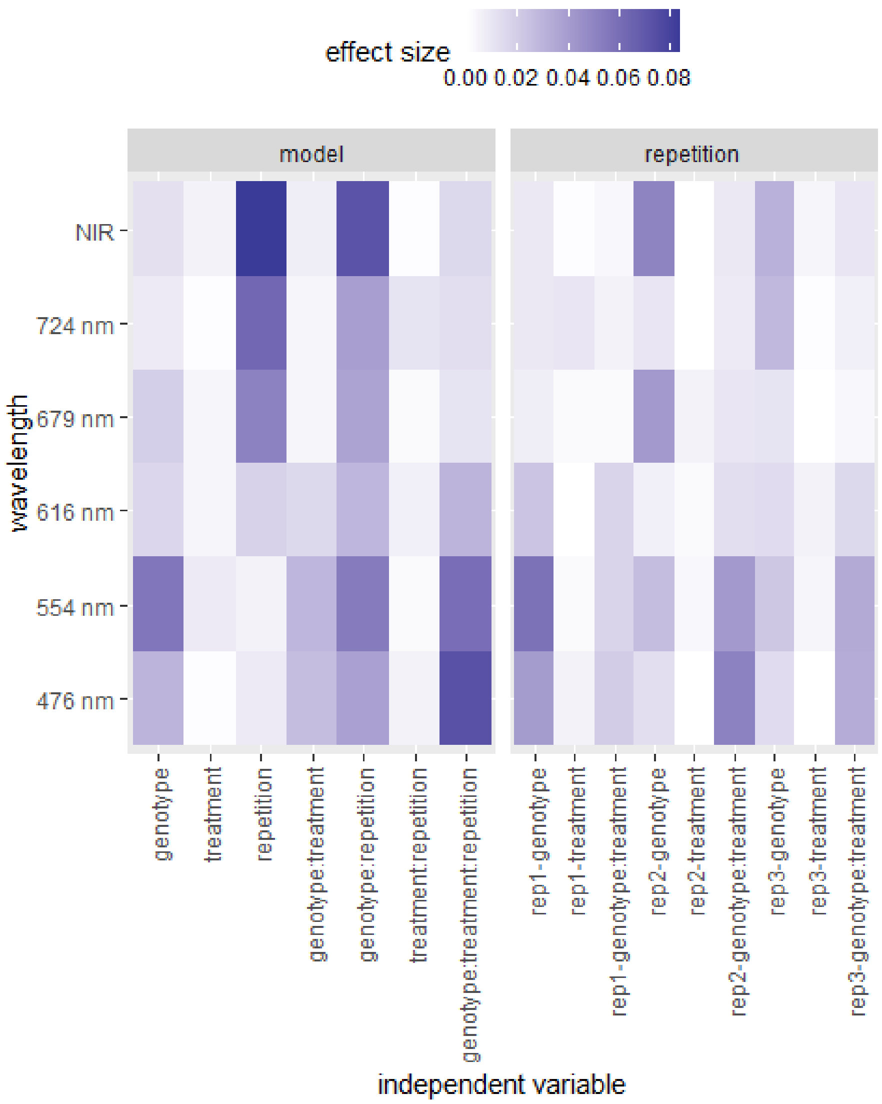

Figure S1.2. Heat map of effect sizes of independent variables of the three-way ANOVA ('model') and the two-way ANOVA per repetition ('repetition') for selected wavelengths calculated at the inflorescence visible stage for nine coastal lowland self-pollinated lines and commercial cv Regalona grown in a field experiment under two treatments (full and reduced irrigation) in three repetitions.

Table S1.1. Effect sizes associated with pairwise genotype comparisons and treatment contrasts for selected wavelengths in three repetitions. Hyperspectral data was acquired for nine coastal lowland self-pollinated lines (CLS) and commercial cv Regalona at the inflorescence visible stage in a field experiment under full irrigation (FI) and reduced irrigation (RI); (a) FI-FI contrasts; (b) RI-RI contrasts; (c) FI-RI contrasts. Only effect sizes >0.5 standard deviation (in absolute values) are reported. The full set can be consulted in File S2.

(a)

Genotype contrast	Treatment contrast	476 nm			554 nm			616 nm			724 nm		
		R1	R2	R3	R1	R2	R3	R1	R2	R3	R1	R2	R3
CLS-1 - CLS-9	FI - FI				-0.60	0.90	-1.10						
CLS-2 - CLS-4	FI - FI							0.76	-0.63	0.63			
CLS-3 - CLS-5	FI - FI										-0.67	-0.53	0.88
CLS-3 - CLS-9	FI - FI	0.52	0.98	-0.55									
CLS-6 - CLS-9	FI - FI	-0.70	0.55	-0.63	-1.28	0.78	-0.99						
CLS-7 - CLS-1	FI - FI				0.82	-0.52	1.11						
CLS-7 - CLS-4	FI - FI							1.28	-0.66	0.85			
CLS-7 - CLS-6	FI - FI	0.84	-0.88	1.04									
CLS-7 - Regalona	FI - FI	1.05	-0.76	1.31	0.92	-1.00	1.10	0.91	-0.69	0.62			
CLS-8 - CLS-4	FI - FI							1.24	-0.50	0.68			
CLS-9 - Regalona	FI - FI				0.70	-1.38	1.08						

The table does not include any results for 679 nm and NIR as no effect sizes >0.5 standard deviation (in absolute values) were found.

(b)

Genotype contrast	Treatment contrast	476 nm			554 nm			NIR		
		R1	R2	R3	R1	R2	R3	R1	R2	R3
CLS-1 - CLS-4	RI - RI	0.93	-0.84	-0.97						
CLS-2 - CLS-5	RI - RI	0.62	-0.71	0.59						
CLS-2 - CLS-8	RI - RI	0.62	-0.97	0.75	0.76	-0.76	1.27			
CLS-2 - CLS-9	RI - RI	0.64	-0.53	0.84						
CLS-6 - Regalona	RI - RI							0.50	0.94	-1.08

The table does not include any results for 616 nm, 679 nm and 724 nm as no effect sizes >0.5 standard deviation (in absolute values) were found.

(c)

Genotype contrast	Treatment contrast	476 nm			554 nm			616 nm			724 nm			NIR		
		R1	R2	R3	R1	R2	R3	R1	R2	R3	R1	R2	R3	R1	R2	R3
CLS-2 - CLS-4	FI - RI	0.99	-0.81	-1.20												
CLS-2 - CLS-8	FI - RI										-0.61	0.67	0.53			
CLS-3 - CLS-1	FI - RI										-0.55	-0.52	0.70			
CLS-3 - CLS-2	FI - RI	0.64	1.35	-0.72												
CLS-3 - CLS-5	FI - RI										-0.86	-0.72	0.93			
CLS-3 - CLS-6	FI - RI										-0.98	-0.60	1.11	-0.81	-0.82	0.62
CLS-3 - CLS-9	FI - RI										-0.85	-0.51	0.72			
CLS-6 - CLS-2	FI - RI	-0.58	0.91	-0.80	-1.16	0.77	-1.04									
CLS-6 - CLS-3	FI - RI													0.54	0.60	-0.64
CLS-7 - CLS-3	FI - RI				1.36	0.71	1.40									
CLS-7 - CLS-5	FI - RI	0.88	-0.67	0.84				0.79	-0.81	0.72						
CLS-7 - CLS-6	FI - RI							0.78	-0.60	0.76						
CLS-7 - CLS-8	FI - RI	0.89	-0.94	0.99												
CLS-7 - CLS-9	FI - RI							0.81	-0.52	0.68						
CLS-8 - CLS-3	FI - RI				1.20	0.52	0.57									
CLS-8 - CLS-4	FI - RI	0.83	-0.93	-0.92												
CLS-8 - CLS-5	FI - RI							0.75	-0.65	0.55						
CLS-9 - CLS-5	FI - RI							0.57	-0.52	0.95						
CLS-9 - CLS-8	FI - RI	0.75	-0.60	0.57	0.88	-0.76	1.23									
CLS-9 - Regalona	FI - RI	0.78	0.53	0.65												
Regalona - CLS-2	FI - RI	-0.79	0.79	-1.07	-0.58	1.37	-1.13									

The table does not include any results for 679 nm as no effect sizes > 0.5 standard deviation (in absolute values) were found.
