# Supplementary material for: Morphological and Physiological Traits Associated with Yield under Reduced Irrigation in Chilean Coastal Lowland Quinoa
Source: Plants (Basel). 2022 Jan 26;11(3):323. doi: 10.3390/plants11030323 (PMC8839172; doi:10.3390/plants11030323)
Supplement: Supplementary file 1 [file plants-11-00323-s001.zip › File S4. Morphological and physiological traits at the inflorescence visible stage.pdf]

S4. Plant phenology, and morphological and physiological traits measured at the inflorescence visible stage

S4.1. Materials and Methods

Plant morphology and biomass

At the inflorescence visible stage (extended BBCH 59 [39]), two plants were randomly selected from each of three plots per genotype and treatment, and excavated. Plant height was measured with a ruler. Leaves were removed from the main plant stem and stored in a paper bag. The maximum stem diameter was determined using a caliper and the stem was subsequently separated from the root crown and stored in a second paper bag. Leaf and stem samples were dried in an oven at 70 °C until a constant weight was reached. Leaf and stem dry weight was determined with a precision balance (BEL Engineering model M214 Ai, Santiago de Chile, Chile). Outliers were identified by applying the interquartile range method (R package 'rstatix' [104]).

Leaf relative water content

To determine relative water content, an approximately 1 cm^3 section of leaf was sampled from each plot and transferred to a pre-weighed Eppendorf tube. The tubes were then reweighed, filled with MilliQ water and left at 4 °C overnight. The following morning, the turgid leaf section was removed from the tube, carefully dried with a paper towel and reweighed. Samples were then placed back into the emptied Eppendorf tubes and placed in an oven at approximately 70 °C overnight. Once leaf sections were completely dry, the tubes containing the leaf samples were reweighed. The empty tube weight was subtracted from the fresh weight and dried weight measurements and relative water content (RWC, %) was calculated using the equation:

$$RWC = \frac{fresh\ weight - dry\ weight}{turgid\ weight - dry\ weight}\ x\ 100$$

Stem and leaf water potential

To conduct stem water potential measurements in the field, two sun-exposed leaves from each plot were wrapped in aluminum foil for at least one hour before measurements began. The leaves in the aluminum foil were cut from the plant stem, placed into a foil-lined plastic bag and immediately transported to the pressure chamber (Model 600, PMS Instrument Company, Albany, OR, USA). In a similar way, two other sun-exposed leaves were sampled from each plot to measure leaf water potential. Plots were measured in pairs (full irrigation, FI, and the corresponding reduced irrigation, RI, plot). Plots of blocks 1 and 4 were measured 49 days after sowing between 15:00 and 19:00. Plots of blocks 2 and 5 were measured 48 days after sowing between 15:00 and 18:00. Plots of blocks 3 and 6 were measured 50 days after sowing between 10:00 and 13:30.

Chlorophyll fluorescence

Fluorescence data was collected using a MINI-PAM-II fluorometer (Walz Heinz GmbH, Effeltrich, Germany). To conduct measurements, the leaf clip was attached to a leaf and measurements of photosynthetically active radiation (PAR) and the photosynthetic efficiency of photosystem II (PhiPSII) were recorded. This was repeated ~10 times per plot. Each FI plot was followed by its RI plot counterpart. Plots of blocks 1 and 4 were measured 49 days after sowing between 14:45 and 15:45. Plots of blocks 2 and 5 were measured 48 days after sowing between 15:00 and 17:00. All blocks were measured 50 days after sowing between 10:24 and 12:49. The fluorescence kinetics of saturation pulse analyses for measurements were

inspected using WinControl-3 Software (version 3.29-rev.1112) (Walz Heinz GmbH, Effeltrich, Germany). Measurements that did not pass inspection were discarded from subsequent analyses.

PhiPSII values were clustered according to genotype, treatment and PAR level using custom R scripts. PAR was divided into four levels: low (<500 µmol photons m^{-2} s^{-1}), mid-low (500-1000 µmol photons m^{-2} s^{-1}), mid-high (1000-1500 µmol photons m^{-2} s^{-1}) and high (>1500 µmol photons m^{-2} s^{-1}). Agglomerative hierarchical clustering was applied on the scaled PhiPSII means for all plots using the 'agnes' function of the R package 'cluster' [100] with a cut-off of five clusters.

S4.2 Results

Plant phenology

No significant differences were observed in seedling emergence or in the progression of early vegetative stages and flowering between lines and treatments, measured both as days after sowing (DAS) and growing degree days (GDD; File S3). We did observe differences between lines in the duration of the seed maturation stages. From the milky grain stage until physiological maturity, Regalona had the shortest seed maturation time, while it was the longest for CLS-5. It took Regalona 98 DAS and 1321 GDD to reach physiological maturity, whereas CLS-5 took 116 DAS and 1616 GDD. Line CLS-7 required less time to reach the doughy stage (83 DAS, 1079 GDD), while CLS-1 and CLS-6 required more time (95 DAS, 1276 GDD and 91 DAS, 1217 GDD respectively). The overall time needed to reach physiological maturity was however identical when compared to the other lines (105 DAS or 1434 GDD on average). There were no significant RI treatment effects at any developmental stage (File S3).

Plant morphology and biomass

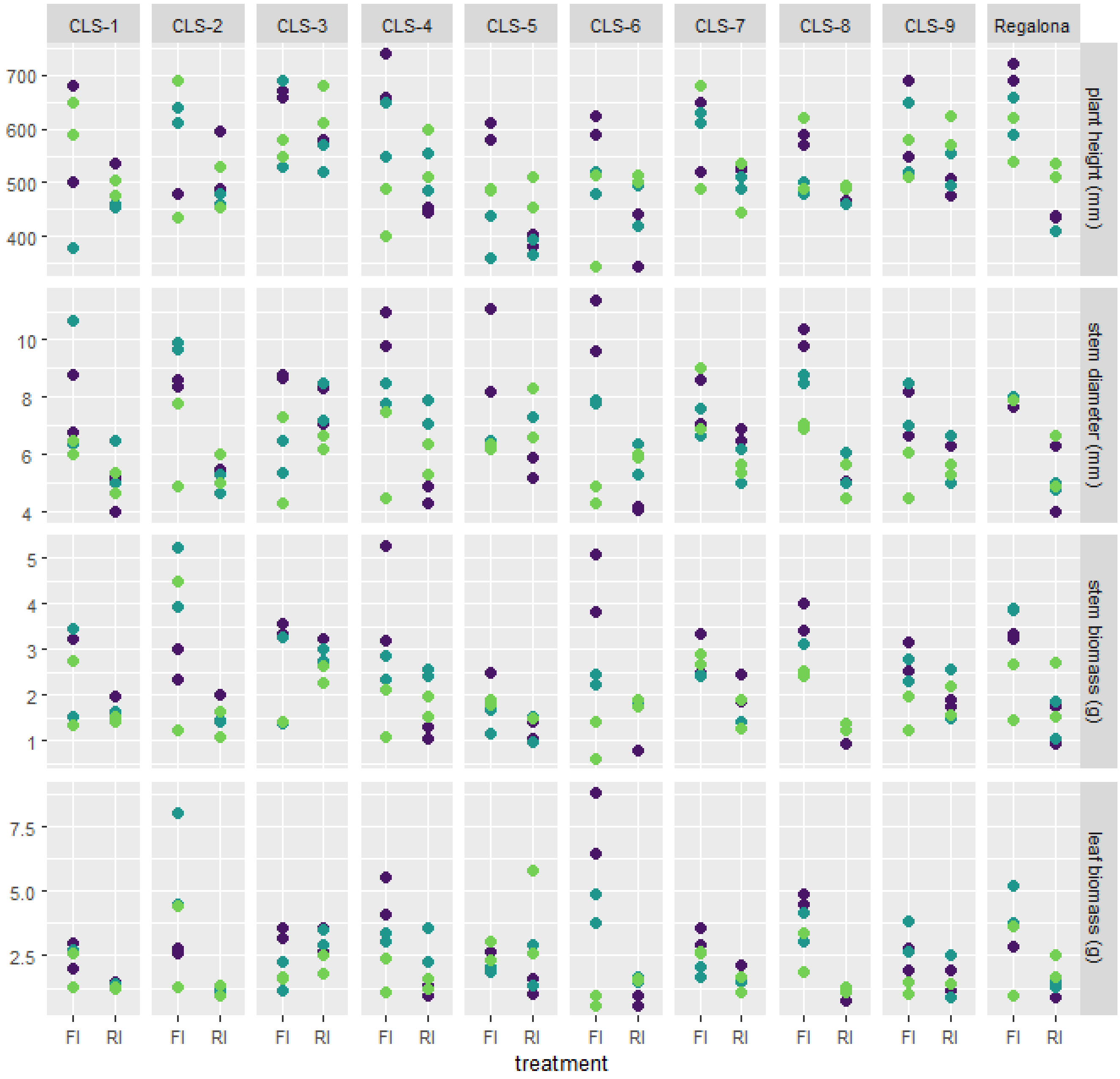

Figure S4.1. Plant height, stem diameter, stem biomass and leaf biomass measured in nine coastal lowland self-pollinated lines (CLS) and commercial cv Regalona at the inflorescence visible stage in a field experiment under full irrigation (FI) and reduced irrigation (RI). Measurements for the plots of blocks 1 and 4, 2 and 5, and 3 and 6 are colored purple, dark green and light green, respectively.

Leaf relative water content

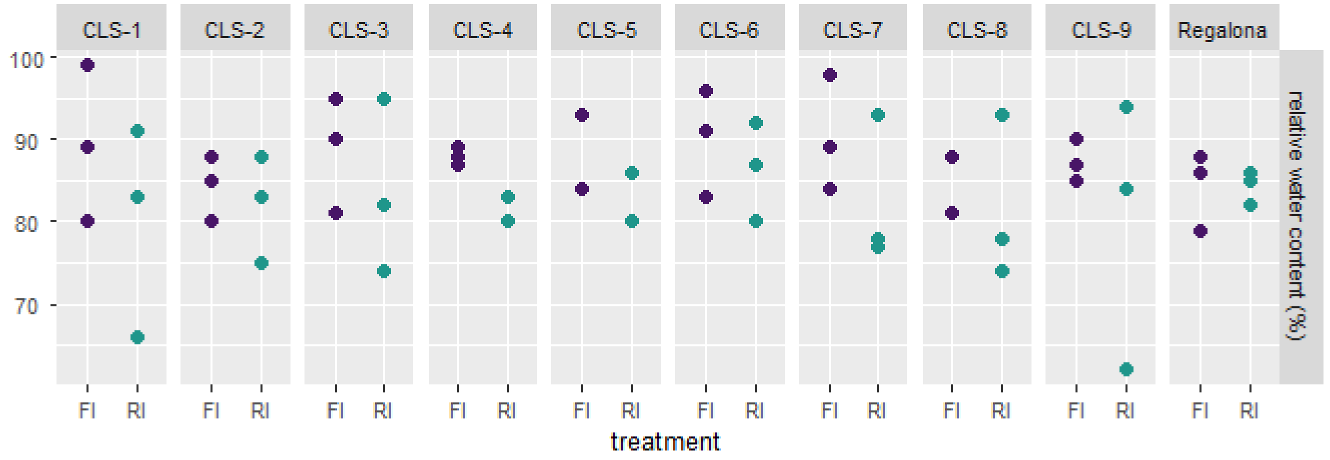

Figure S4.2. Leaf relative water content measured in nine coastal lowland self-pollinated lines (CLS) and commercial cv Regalona at the inflorescence visible stage in a field experiment under full irrigation (FI, purple) and reduced irrigation (RI, green).

Stem and leaf water potential

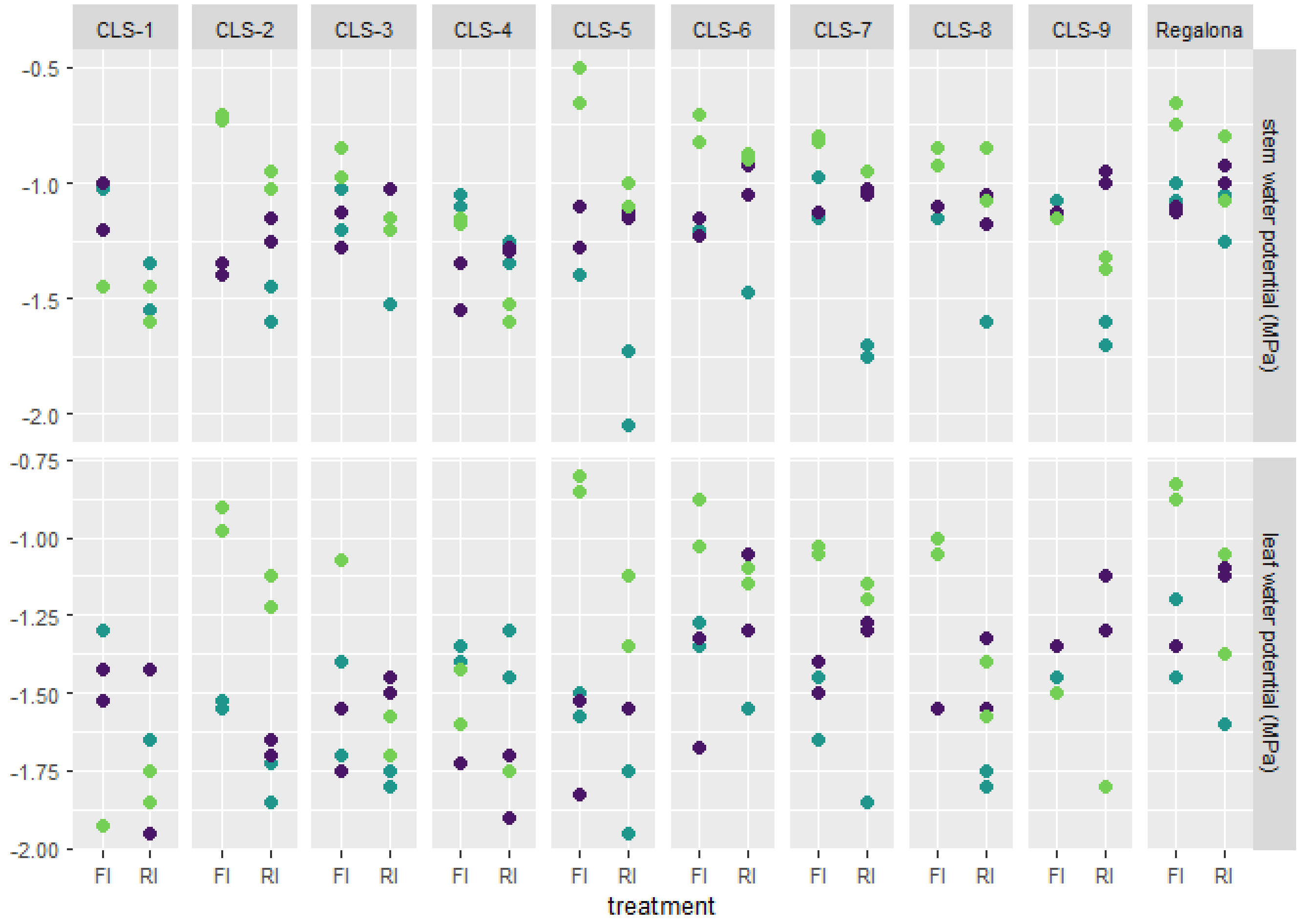

Figure S4.3. Stem and leaf water potential measured in nine coastal lowland self-pollinated lines (CLS) and commercial cv Regalona at the inflorescence visible stage in a field experiment under full irrigation (FI) and reduced irrigation (RI). Measurements for the plots of blocks 1 and 4, 2 and 5, and 3 and 6 are colored purple, dark green and light green, respectively.

Chlorophyll fluorescence

Different patterns of mean quantum efficiency of photosystem II in response to treatment were observed (Figure S4.4). Lines CLS-3 and CLS-5 clustered together for both full irrigation (FI) and reduced irrigation (RI) treatments, while no other lines consistently clustered together for both treatments. This result suggests similar responses to both treatment and PAR level for the two lines. Treatment had the greatest influence on CLS-8, which clustered next to CLS-9 for FI samples, demonstrating the lowest photosynthetic efficiency of all lines at low PAR. In the RI treatment, CLS-8, along with CLS-1 and CLS-6, demonstrated high photosynthetic efficiency at high, mid-low and low PAR compared to the other lines. CLS-9 FI samples generally had low photosynthetic efficiency for all PAR levels compared to other lines, except at low PAR. In CLS-9 RI samples, low photosynthetic efficiency was only observed for high and mid-low PAR.

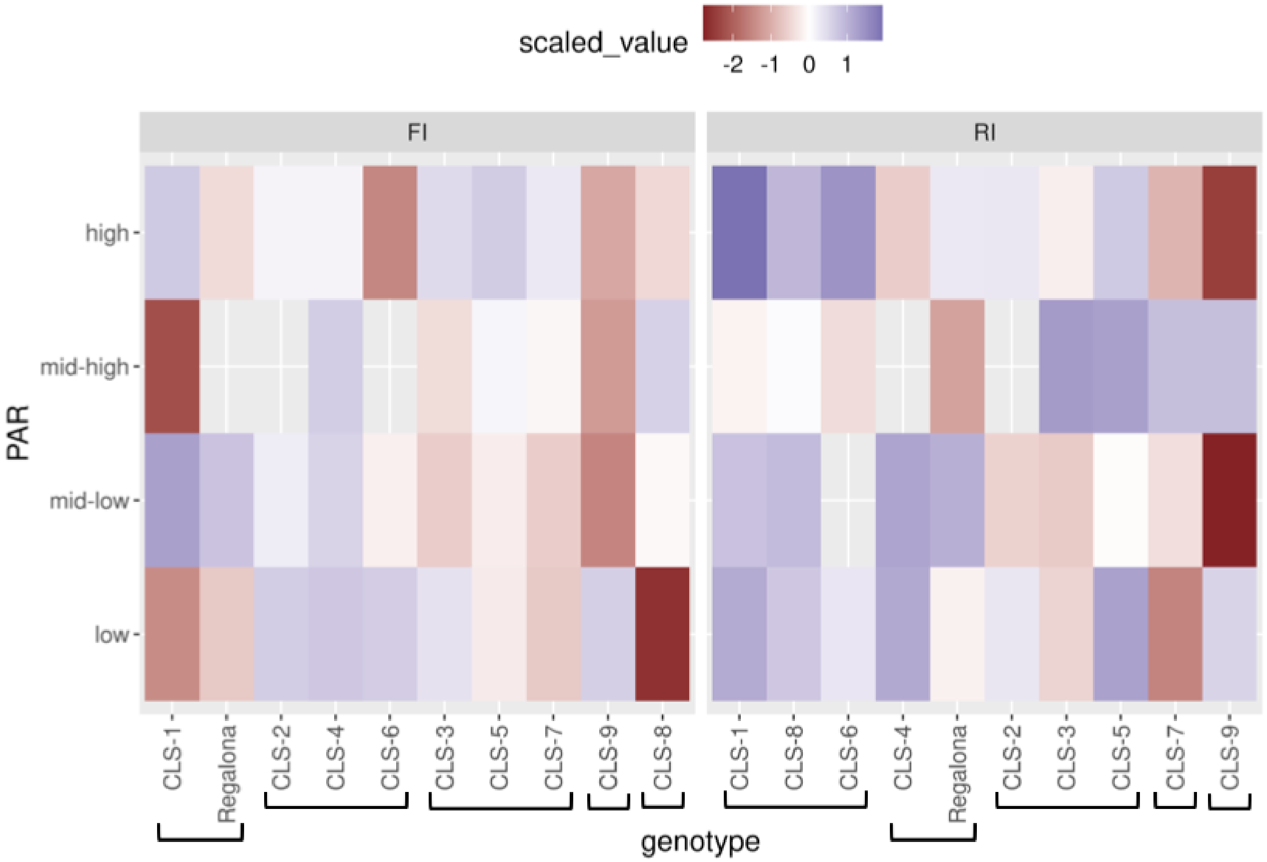

Figure S4.4. Heat map of scaled mean values of the quantum efficiency of photosystem II for nine coastal lowland self-pollinated lines (CLS) and commercial cv Regalona at the inflorescence visible stage in a field experiment under full irrigation (FI) and reduced irrigation (RI). Lines are grouped per cluster for each treatment. For photosynthetically active radiation (PAR) values, low was designated as <500 μmol photons m^{-2} s^{-1}, mid-low as 500-1000 μmol photons m^{-2} s^{-1}, mid-high as 1000-1500 μmol photons m^{-2} s^{-1} and high as >1500 μmol photons m^{-2} s^{-1}.
