# Supplementary material for: Morphological and Physiological Traits Associated with Yield under Reduced Irrigation in Chilean Coastal Lowland Quinoa
Source: Plants (Basel). 2022 Jan 26;11(3):323. doi: 10.3390/plants11030323 (PMC8839172; doi:10.3390/plants11030323)
Supplement: Supplementary file 1 [file plants-11-00323-s001.zip › Table S1. Vegetation indices.pdf]

Table S1. Vegetation indices included in the analysis of quinoa hyperspectral data.

Index	Name	Formula	Sensitivity	Reference
ARI	Anthocyanin Reflectance index	$\frac{1}{R550} - \frac{1}{R700}$	carotenoids	Gitelson et al. [50]
BGI	Blue Green pigment Index	$\frac{R450}{R550}$	dry matter, pigments	Zarco-Tejada et al. [62]
BRI	Blue Red pigment Index	$\frac{R450}{R690}$	dry matter, pigments	Zarco-Tejada et al. [62]
CRI	Carotenoid Reflectance Index	$\frac{1}{R510} - \frac{1}{R550}$	carotenoids	Gitelson et al. [50]
CRI2	Carotenoid Reflectance Index	$\frac{1}{R510} - \frac{1}{R700}$	carotenoids	Gitelson et al. [50]
G	Greenness index	$\frac{R554}{R677}$	chlorophyll	Zarco-Tejada et al. [62]
GM1	Gitelson & Merzlyak index	$\frac{R750}{R550}$	chlorophyll	Gitelson and Merzlyak [51]
GM2	Gitelson & Merzlyak index	$\frac{R750}{R700}$	chlorophyll	Gitelson and Merzlyak [51]
gNDVI	Green Normalized Difference Vegetation Index	$\frac{R750 - R550}{R750 + R550}$	chlorophyll	Datt [46]
hNDVI	Hyperspectral Normalized Difference Vegetation Index	$\frac{R827 - R668}{R827 + R668}$	structure	Oppelt [56]
LCI	Leaf Chlorophyll Index	$\frac{R850 - R710}{R850 - R680}$	chlorophyll	Datt [45]
LIC1	Lichtenthaler Index	$\frac{R800 - R680}{R800 + R680}$	fluorescence	Lichtenthaler et al. [54]
LIC2	Lichtenthaler Index	$\frac{R440}{R690}$	fluorescence	Lichtenthaler et al. [54]
LIC3	Lichtenthaler Index	$\frac{R440}{R740}$	fluorescence	Lichtenthaler et al. [54]
MCARI	Modified Chlorophyll Absorption in Reflectance Index	$[(R700 - R670) - 0.2\ (R700 - R550)]\left(\frac{R700}{R670}\right)$	chlorophyll	Daughtry et al. [47]

Index	Name	Formula	Sensitivity	Reference
MCARI1	Modified Chlorophyll Absorption in Reflectance Index	$1.2\,[2.5\,(R800 - R670) - 1.3\,(R800 - R550)]$	structure	Haboudane et al. [52]
MCARI2	Modified Chlorophyll Absorption in Reflectance Index	$\frac{1.5\,[2.5\,(R800 - R670) - 1.3\,(R800 - R550)]}{\sqrt{(2\,R800 + 1)^2 - \left(6\,R800 - 5\,\sqrt{R680}\right) - 0.5}}$	structure	Haboudane et al. [52]
NDVI	Normalized Difference Vegetation Index	$\frac{NIR - RED}{NIR + RED}$	structure	Rouse et al. [61]
NDVIAparicio	Normalized Difference Vegetation Index	$\frac{R900 - R680}{R900 + R680}$	structure	Aparicio et al. [42]
NDVIHaboudane	Normalized Difference Vegetation Index	$\frac{R800 - R670}{R800 + R670}$	structure	Haboudane et al. [52]
NDVIZarcoTejada	Normalized Difference Vegetation Index	$\frac{R774 - R677}{R774 + R677}$	structure	Zarco-Tejada and Miller [64]
NPCI	Normalized Pigment Chlorophyll Index	$\frac{R680 - R430}{R680 + R430}$	dry matter, pigments	Peñuelas et al. [58]
PRI	Photochemical Reflectance Index	$\frac{R528 - R567}{R528 + R567}$	chlorophyll	Gamon et al. [48]
PRIPenuelas	Photochemical Reflectance Index	$\frac{R531 - R570}{R531 + R570}$	chlorophyll	Peñuelas et al. [57]
PSSRa	Pigment Specific Simple Ratio for chlorophyll a	$\frac{R800}{R675}$	chlorophyll	Blackburn [43]
PSSRb	Pigment Specific Simple Ratio for chlorophyll b	$\frac{R800}{R650}$	chlorophyll	Blackburn [43]
PSSRc	Pigment Specific Simple Ratio for carotenoids	$\frac{R800}{R500}$	carotenoids	Blackburn [43]
RDVI	Renormalized Difference Vegetation Index	$\frac{R800 - R670}{\sqrt{R800 + R670}}$	structure	Roujean and Breon [60]
RGI	Red Green pigment Index	$\frac{R690}{R550}$	dry matter, pigments	Zarco-Tejada et al. [62]
RGRI	Red Green Ratio Index	$\frac{RRED}{RGREEN}$	structure	Rouse et al. [61]; Gamon and Surfus [49]

Index	Name	Formula	Sensitivity	Reference
SIPI	Structure Insensitive Pigment Index	$\frac{R800 - R445}{R800 - R680}$	pigments	Peñuelas et al. [57]
SR	Simple Ratio index	$\frac{RNIR}{RRED}$	structure	Jordan [53]
SRChl	Simple Ratio index for Chlorophyll	$\frac{R672}{R550\ x\ R700}$	chlorophyll	Datt [46]
SRChla	Simple Ratio index for Chlorophyll a	$\frac{R675}{R700}$	chlorophyll	Datt [46]
SRChlb	Simple Ratio index for Chlorophyll b	$\frac{R675}{R650\ x\ R700}$	chlorophyll	Datt [46]
SRChlb2	Simple Ratio index for Chlorophyll b	$\frac{R672}{R708}$	chlorophyll	Datt [46]
SRChltot	Simple Ratio index for total Chlorophyll	$\frac{R760}{R500}$	chlorophyll	Datt [46]
TVI	Triangular Vegetation Index	$0.5\ [120\ (R750 - R550) - 200\ (R670 - R550)]$	chlorophyll	Broge and Leblanc [44]
WBI	Water Band Index	$\frac{R970}{R900}$	water	Peñuelas et al. [59]
WCI	Water Content Index	$\frac{(R686 - R955)}{(R955 - R548)}$	water	Mertens et al. [55]
ZTM	Zarco-Tejada & Miller	$\frac{R750}{R710}$	chlorophyll	Zarco-Tejada et al. [63]
