# Supplementary material for: Morphological and Physiological Traits Associated with Yield under Reduced Irrigation in Chilean Coastal Lowland Quinoa
Source: Plants (Basel). 2022 Jan 26;11(3):323. doi: 10.3390/plants11030323 (PMC8839172; doi:10.3390/plants11030323)
Supplement: Supplementary file 1 [file plants-11-00323-s001.zip › Table S2. Correlations between traits and vegetation indices.pdf]

Table S2. Correlation coefficients and coefficients of determination (R/R^2) of the relation between morphological, physiological and performance traits measured at the inflorescence visible stage (no shading) and at harvest (gray shading), and vegetation indices in nine coastal lowland self-pollinated lines and commercial cv Regalona grown in a field experiment under full irrigation and reduced irrigation. Only the R and R^2 of significant correlations ($p < 0.05$) is shown.

**trait**	**ARI**	**BGI**	**BRI**	**CRI**	**CRI2**	**G**	**GM2**	**GMI**	**gNDVI**	**hNDVI**	**LCI**	**LIC1**	**LIC2**	**LIC3**
plant height			0.34 /0.12									0.28 /0.08	-0.37 /0.14	
stem diameter			0.29 /0.08										-0.30 /0.09	-0.32 /0.10
leaf biomass														
stem biomass			0.33 /0.11										-0.33 /0.11	-0.29 /0.08
leaf relative water content	-0.31 /0.09				-0.29 /0.08	0.28 /0.08		0.27 /0.07	-0.27 /0.07					
stem water potential		-0.43 /0.19	-0.28 /0.08	0.43 /0.18								0.35 /0.12	0.34 /0.12	0.59 /0.34
leaf water potential		-0.42 /0.17	-0.27 /0.07	0.34 /0.11								0.26 /0.07	0.35 /0.12	0.56 /0.31
thermal index 1				0.29 /0.08						0.32 /0.10		0.39 /0.16		0.37 /0.14
plant height		0.35 /0.12	0.41 /0.17	-0.27 /0.07			-0.42 /0.17			0.27 /0.07	0.42 /0.18		-0.52 /0.27	-0.40 /0.16
yield														
1000 seed weight														-0.26 /0.07
1.7 mm caliber seed weight														
1.4 mm caliber seed weight														
1.18 mm caliber seed weight														
panicle length							-0.30 /0.09				0.34 /0.11			
panicle width														

Table S2. Continued.

trait	MCARI	MCARI1	MCARI2	NDVI	NDVIAparicio	NDVIHaboudane	NDVIZarco-Tejada	NPCI	PRI	PRIPenuelas	PSSRa	PSSRb	PSSRc
plant height						0.26 /0.07		-0.33 /0.11			0.26 /0.07		
stem diameter								-0.29 /0.08					
leaf biomass								-0.26 /0.07					
stem biomass								-0.29 /0.09					
leaf relative water content			0.27 /0.07										-0.28 /0.08
stem water potential	0.26 /0.07				0.38 /0.14	0.29 /0.08	0.35 /0.13	0.41 /0.17	-0.62 /0.38	-0.55 /0.30	0.33 /0.11		
leaf water potential	0.29 /0.09				0.29 /0.08		0.27 /0.07	0.35 /0.12	-0.53 /0.28	-0.47 /0.22			
PSII efficiency_low light	-0.51 /0.26	-0.44 /0.19							0.40 /0.16	0.39 /0.16			
thermal index 1					0.40 /0.16	0.35 /0.13	0.38 /0.14		-0.40 /0.16	-0.34 /0.11	0.37 /0.14		
plant height	-0.34 /0.12							-0.51 /0.26		0.28 /0.08		0.26 /0.07	
yield													
1000 seed weight								-0.27 /0.07					
1.7 mm caliber seed weight													
1.4 mm caliber seed weight													
1.18 mm caliber seed weight													
panicle length	-0.29 /0.08	0.27 /0.07								0.25 /0.06			
panicle width													

Table S2. Continued.

trait	RDVI	RGI	RGRI	SIPI	SR	SRChl	SRChla	SRChlb	SRChlb2	SRChltot	TVI	WBI	WCI	ZTM
plant height				-0.42 /0.18										
stem diameter				-0.31 /0.09										
leaf biomass												-0.26 /0.07		
stem biomass				-0.36 /0.13										
leaf relative water content		-0.31 /0.10	-0.33 /0.11							-0.29 /0.09			-0.27 /0.07	
stem water potential				0.29 /0.09			-0.33 /0.11		-0.42 /0.18			0.28 /0.08		
leaf water potential				0.30 /0.09			-0.32 /0.10		-0.37 /0.14					
PSII efficiency_low light						0.46 /0.21	0.41 /0.17	0.47 /0.22	0.37 /0.14		-0.45 /0.20			
thermal index 1												0.33 /0.11		
plant height				-0.45 /0.21			0.43 /0.19	0.30 /0.09	0.37 /0.14			-0.29 /0.09		0.42 /0.18
yield														
1000 seed weight				-0.28 /0.08										
1.7 mm caliber seed weight												-0.26 /0.07		
1.4 mm caliber seed weight														
1.18 mm caliber seed weight												0.29 /0.08		
panicle length	0.33 /0.11						0.31 /0.09		0.31 /0.10		0.28 /0.08	-0.42 /0.17		0.30 /0.09
panicle width												-0.31 /0.10		
